# Genomic Signatures of Selection Are Enriched in Differentially Expressed Genes in Sticklebacks Adapting to Contrasting Environments

**DOI:** 10.1093/gbe/evag222

**Published:** 2026-08-30

**Authors:** Alexander Kwakye, Natalie Dzikowski, Matthew A Wund, Krishna R Veeramah

**Affiliations:** Department of Ecology and Evolution, Stony Brook University, Stony Brook, NY 11794, USA; The Graduate Program in Genetics, Stony Brook University, Stony Brook, NY 11794, USA; Department of Molecular Genetics and Microbiology, Duke University, Durham, NC 27710, USA; University Program in Genetics and Genomics, Duke University, Durham, NC 27710, USA; Department of Biology, The College of New Jersey, Ewing, NJ 08628, USA; Department of Ecology and Evolution, Stony Brook University, Stony Brook, NY 11794, USA

**Keywords:** Threespine stickleback (*Gasterosteus aculeatus*), Genotype–phenotype mapping, Differential gene expression, Freshwater adaptation, Cis-regulation

## Abstract

Whole genome scans have identified numerous adaptive alleles in many species; however, linking these alleles to specific phenotypes remains a major challenge. A promising alternative to direct genotype–phenotype mapping, particularly given the complexities introduced by epistasis, pleiotropy, and environmental variability, is to assess whether differentially expressed genes (DEGs) are enriched in regions of genetic divergence between populations adapted to contrasting environments. Here, we study gene expression patterns in threespine stickleback populations adapting to contrasting environments (marine vs. freshwater) and investigate signatures of selection associated with gene expression evolution during adaptation. We performed transcriptomic experiments of the brain and gill tissues of wild-caught sticklebacks sampled from one marine and two freshwater environments using TagSeq. We found that DEGs in the freshwater environments harbor single nucleotide polymorphisms (SNPs) previously identified to be involved in rapid adaptation and F*_ST_* outliers. A majority of these SNPs were located in cis-regulatory regions of the genes with predicted low to moderate effects on protein function and structure, although we found a high-impact SNP in the gene *col8a1b*. Genes such as *pvalb4* and *acsl4a*, involved in calcium regulation in the gill and fatty acid metabolism in the brain, respectively, were enriched with SNPs showing signatures of selection. By linking signatures of selection to tissue-specific gene expression patterns, our study bridges the gap between genomic divergence and the molecular mechanisms underlying physiological adaptation to new environments and identifies specific pathways that can be targeted for future functional studies.

Significance The molecular basis of adaptive trait divergence is central to evolutionary biology. Whole genome scans have identified numerous candidate alleles underlying adaptive traits, but connecting these alleles to functionally relevant phenotypes is hampered by epistasis, pleiotropy, and environmental effects. By integrating previously identified single nucleotide polymorphisms with tissue-specific gene expression changes, we identify specific SNPs associated with genes involved in physiological processes such as ion regulation in gills and fatty acid metabolism in the brain during adaptation to freshwater environments by oceanic sticklebacks. These SNPs and their associated differentially expressed genes could serve as the basis for functional genomic analyses to further understand phenotypic divergence during freshwater adaptation.

## Introduction

In the last 25 years, the emergence of genomic-scale data has enabled the discovery of numerous loci across many species that demonstrate signals consistent with positive selection (Kerem et al. 1989; Colosimo et al. 2004; Linnen et al. 2009; Jones et al. 2012; Radwan and Babik 2012; Lamichhaney et al. 2015; Massey and Wittkopp 2016; Roberts Kingman et al. 2021; Bomblies and Peichel 2022; Campagna and Toews 2022; Moreira and Smith 2023; Hao et al. 2024; Peng et al. 2024; Yang et al. 2024). While some of these loci have been shown to also have large phenotypic effects (Colosimo et al. 2004; Linnen et al. 2009; Lamichhaney et al. 2015, 2016), in most cases, the traits influenced by adaptive alleles have remained unidentified. Theoretical models have long suggested that adaptation is often driven by many alleles of small effects (Fisher 1958, 1999; Kimura 2012). More recent empirical work, particularly QTL-based studies, have frequently identified loci of moderate to large effect (Colosimo et al. 2005; Hoekstra et al. 2006; Fang et al. 2020; Schluter et al. 2021), though such loci may be disproportionately detectable relative to small-effect variants (Rockman 2012). Consequently, a major challenge moving forward is to link these putative adaptive alleles of small effect to the phenotypes they influence.

Mapping genotypes to specific phenotypes, especially alleles of small effect, remains daunting for several reasons. First, environmental variables can substantially influence gene expression, altering the relationship between phenotype and the underlying genetic variation. Furthermore, the genetic background can modulate an allele's effect through mechanisms such as epistasis (Stern et al. 2022) or pleiotropy (McKay et al. 2003; Rennison and Peichel 2022). Another layer of complexity lies in how phenotypes are defined (Ahnert 2017); phenotypes may encompass amino acids or the protein residues they encode, as well as broader molecular pathways, physiological processes, or morphological traits that arise from these molecular entities or processes. The more hierarchical levels separating the genotype and associated phenotype of interest, the more complex the mapping relationship is likely to be. In addition, large sample sizes are required to detect alleles of small effects when conducting QTL studies (Mackay et al. 2009).

A promising approach that can help sidestep some of these complexities is to estimate whether DEGs are enriched in regions of genetic divergence between populations inhabiting contrasting environments (Verta and Jones 2019; Mack et al. 2023; Forsberg et al. 2025; Rodríguez-Ramírez and Peichel 2025). This approach is based on an assumption that genetic signatures of adaptation found in or nearby DEGs are involved in gene expression divergence between populations in contrasting environments. By design, this approach targets variants found in gene bodies and cis-regulatory regions, rather than trans-regulatory variants or even more distantly acting cis-regulatory variants that may be in linkage disequilibrium with target variants. Thus, this approach cannot capture all genetic signatures of adaptation that influence gene expression. However, it is particularly well suited for systems where alleles at many adaptive loci act primarily through cis-regulatory changes, such as those proposed to be involved in freshwater adaptation in threespine stickleback (Jones et al. 2012; Verta and Jones 2019; Mack et al. 2023).

Threespine stickleback (*Gasterosteus aculeatus*) is an ancestral marine (Bell et al. 2009) fish that is found along the coastal regions of the Northern hemisphere. There are many ecotypes of this fish, which are often considered to reside in pairs of contrasting environments, such as marine–freshwater, benthic–limnetic, and lake–stream habitats (Reid et al. 2021). The marine ecotype has repeatedly adapted to new freshwater environments for at least the past 10 million years (Bell and Foster 1994). Adaptation to freshwater environments is characterized by rapid phenotypic changes, which are observed within a few generations (Heuts 1947; Bell and Richkind 1981; Bell et al. 2004), as well as rapid genomic divergence (Colosimo et al. 2004; Cresko et al. 2004; Jones et al. 2012; Marchinko et al. 2014; Roberts Kingman et al. 2021; Kwakye et al. 2026). There are more than 300 loci in the genome that have alleles that rapidly increase in frequency when marine fishes are introduced into freshwater environments (Hohenlohe et al. 2012; Jones et al. 2012; O’Brown et al. 2015; Roberts Kingman et al. 2021). However, the phenotypic effects and functional roles during freshwater adaptation for most of these alleles are largely unknown, with the exception of a handful of loci such as *EDA* (Colosimo et al. 2004).

The gill and brain play important roles in physiological and behavioral phenotypes involved in adaptation to new freshwater environments by marine threespine stickleback (Di Poi et al. 2016). The gill is a key osmoregulatory organ important for transitions from marine to freshwater environments (Bonga 1973; Judd 2012; Hasan et al. 2017). Osmoregulation is fundamental during the early stages of freshwater adaptation (McCairns and Bernatchez 2010; Divino et al. 2016; Kusakabe et al. 2019). Adaptation to freshwater environments by marine populations is also characterized by marked behavioral changes mediated by structures in the brain (Park and Bell 2010; Bensky and Bell 2022). When threespine sticklebacks from a freshwater environment were exposed to predation risk, selection favored aggressive individuals (Bell and Sih 2007). There are also heritable differences in schooling behavior between marine and freshwater stickleback (Wark et al. 2011; Greenwood et al. 2013).

Using F1 marine–freshwater hybrids of threespine stickleback, Mack et al. (2023) showed that genes with allele-specific expression in multiple tissues (including the brain and gill) are enriched in regions of genomic divergence between marine and freshwater populations. Differentially spliced genes between marine and freshwater ecotypes of threespine sticklebacks have also been found to be enriched in these genomic regions of marine–freshwater divergence (Rodríguez-Ramírez and Peichel 2025). However, it remains unclear to what extent these gene expression changes reflect those observed in nature. Most studies that have quantified gene expression changes in the gill have used lab-raised threespine stickleback (Verta and Jones 2019; Mack et al. 2023; Rodríguez-Ramírez and Peichel 2025). Laboratory conditions control biological noise but eliminate the influence of environmental variability, which is a crucial factor that affects genotype–phenotype relationships. Environmental volatility can also be impactful in the molecular processes that allow an organism to adapt to its local habitat. Additionally, studies from Mack et al. (2023), Rodríguez-Ramírez and Peichel (2025), and Verta and Jones (2019) focused on the enrichment of DEGs within genomic regions of divergence between marine and freshwater populations that each consists of up to thousands of SNPs and which can sometimes overlap multiple genes (Jones et al. 2012; Roberts Kingman et al. 2021). While multiple studies have directly measured gene expression changes in the gills of marine and freshwater sticklebacks, there have also been very limited studies that specifically compare gene expression changes in the brain of marine and freshwater stickleback except for two microarray studies (Ishikawa et al. 2017; Kitano et al. 2019).

To complement these studies that focused on lab-raised sticklebacks, we examined gene expression patterns in the gill and brain of wild-caught threespine sticklebacks sampled from one marine and two freshwater populations. The marine samples were collected from Rabbit Slough, a freshwater stream that drains into a nearby estuary where sticklebacks migrate from the ocean to breed in the summer. One of the freshwater populations (Cheney Lake) has only recently diverged from a marine progenitor (Bell et al. 2016; Aguirre et al. 2022), while the other (Cornelius Lake) is putatively a long-established population. Unlike previous studies that examined whole regions, our analytical framework focused on SNPs associated with the DEGs which allowed us to identify specific nucleotide changes that may be mediating certain crucial biological processes for freshwater adaptation, which could be directly targeted for future functional analyses.

## Results

### Population Sampling and Demography

**Fig. 1.**
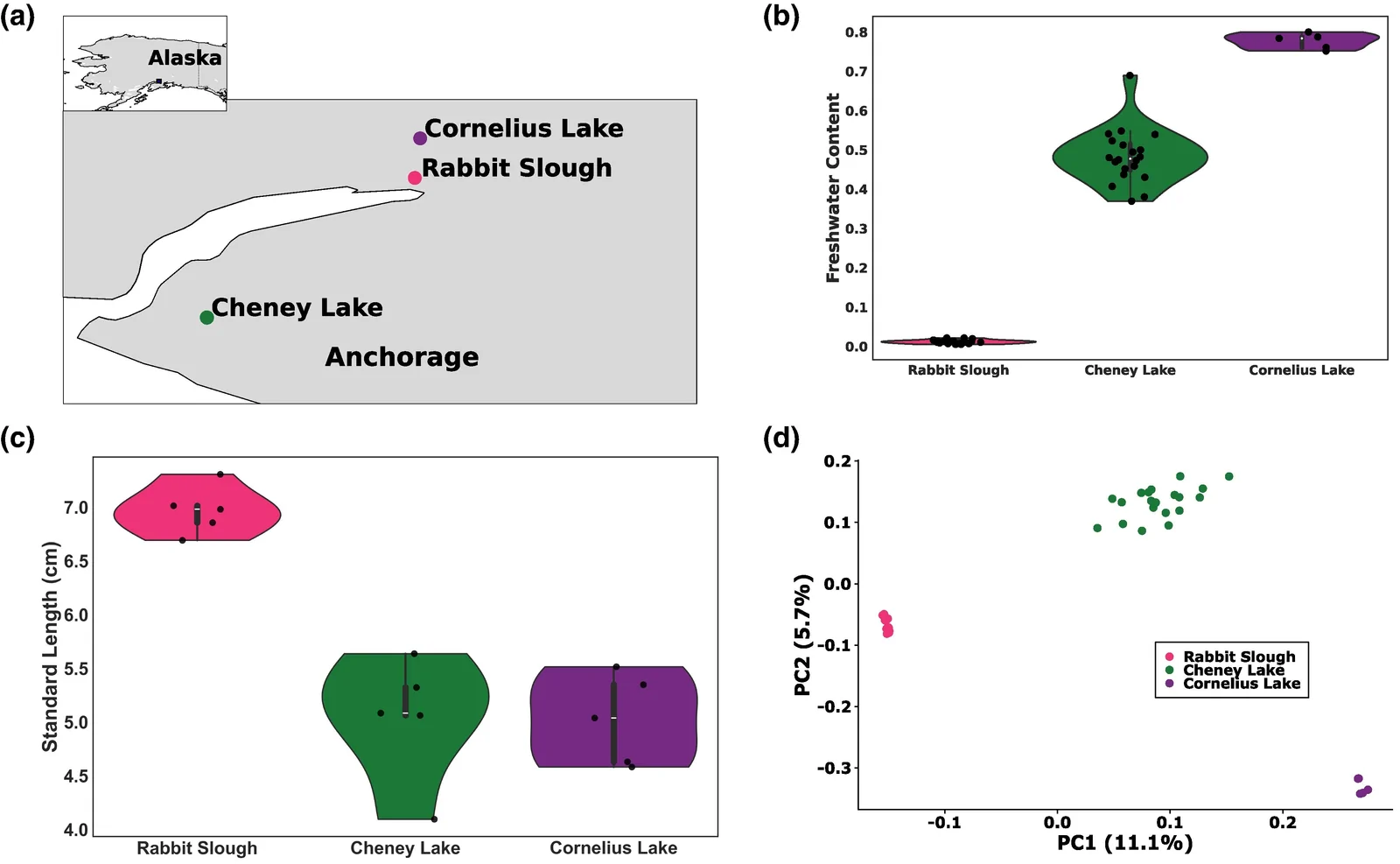
a) Location of lakes used in this study. b) Proportion of freshwater-adaptive alleles per individual for samples collected from the three environments. c) Standard lengths of samples used for RNA sequencing. d) Principal components analysis (PCA) using genome-wide SNPs.

We collected brain and gill tissues from threespine stickleback individuals from three distinct Alaskan populations in the Anchorage/Matanuska–Susitna Valley region: Rabbit Slough, Cheney Lake, and Cornelius Lake (Methods, Fig. 1a). Cheney is approximately 60 km south of Rabbit Slough, while Cornelius is 13 km north of it. We sampled five individuals from each population and generated TagSeq gene expression datasets from gill and brain tissues (total *n* = 30, Methods). The sampled individuals included three females and two males from Rabbit Slough; three males and two females from Cheney Lake; and two females and three males from Cornelius Lake. Rabbit Slough is an anadromous population that has been found in many new freshwater populations including Cheney Lake (Bell et al. 2016; Aguirre et al. 2022). In 2009, 3,000 anadromous individuals from Rabbit Slough were transplanted into Cheney Lake in order to reestablish the stickleback population following their extirpation, which resulted from an effort to eliminate invasive northern pike (*Esox lucius*) from the lake. Previous phenotypic and genomic studies revealed rapid adaptation to this new freshwater environment (Wund et al. 2016; Roberts Kingman et al. 2021). The average standard length of Rabbit Slough specimens was 7 cm, consistent with most marine threespine sticklebacks, whereas specimens from Cheney and Cornelius Lakes had standard lengths of 5.04 cm and 5.02 cm, respectively, characteristic of mature freshwater sticklebacks (Baker 1994; DeFaveri and Merilä 2013) (Fig. 1c).

**Fig. 2.**
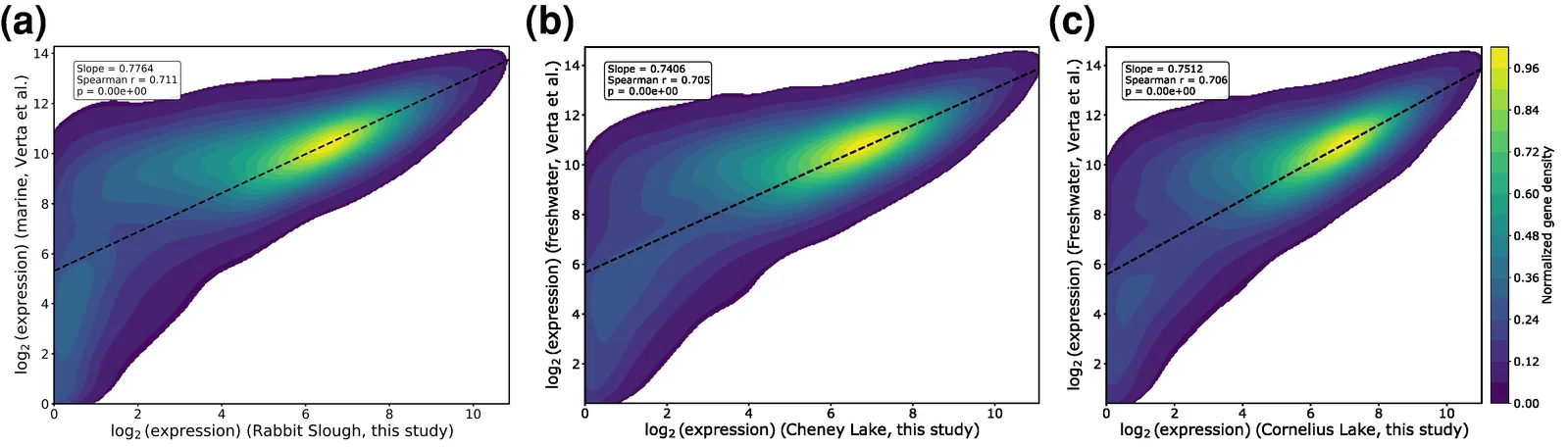
Comparison of bulk RNAseq dataset from Verta and Jones (2019) to TagSeq dataset in the present study. Correlation of gene counts estimated using TagSeq for a) Rabbit Slough individuals versus marine individuals collected from Little Campbell River; b) Cheney Lake individuals versus freshwater individuals collected from Little Campbell River; c) Cornelius individuals versus freshwater individuals collected from Little Campbell River.

The threespine stickleback population found in Cornelius Lake has been putatively established for a much longer time than Cheney (assumed to be several thousand years); however, no previous study has determined the demographic history of this threespine stickleback population. Therefore, in order to explore its population genetic structure, we performed whole genome sequencing (WGS) for five Cornelius Lake specimens to approximately 6× coverage. We used an approximate genotype likelihood approach to call the diploid state of alleles at freshwater-adaptive loci previously identified by Roberts Kingman et al. (2021) as homozygous oceanic, heterozygous, or homozygous freshwater. This approach accurately identifies genotypes at genomic data at coverages as low as 0.4× (Kwakye et al. 2026), allowing us to effectively call freshwater/marine genotype status for the Cornelius Lake specimens which were sequenced at 6X. The genotypes at these loci were predominantly freshwater (Fig. S1). On average, each individual had ∼75% freshwater content, which reflects what is observed in other established freshwater populations in the Pacific Northwest (Fig. 2b, Fig. S2). In comparison, the average freshwater content in Rabbit Slough (based on *n* = 20, 20× WGS published in Roberts Kingman et al. (2021)) and Cheney Lake (based on *n* = 20, 30× WGS published in Kwakye et al. (2026)) was 1.2% and 48%, respectively (Fig. 2b).

We used principal component analysis (PCA) and pairwise *F_ST_* to determine the population genetic structure among the three populations used in this study. PC1 separated the samples based on population, with Cheney being intermediate between Rabbit Slough, from which it was recently derived, and Cornelius Lake, which is an established freshwater population (Fig. 1d). Our estimates of pairwise *F_ST_* using SNPs from the WGS showed similar patterns of population differentiation, with a mean genome-wide *F_ST_* of 0.033 between Rabbit Slough and Cheney Lake samples; 0.084 between Rabbit Slough and Cornelius Lake samples; and 0.07 between Cheney Lake and Cornelius Lake samples.

We then estimated *F_ST_* using a set of SNPs with putatively freshwater-adaptive alleles that were identified in Roberts Kingman et al. (2021), called tempo SNPs. Tempo SNPs are found within genomic regions containing genetic variation that was previously shown to experience rapid and significant coordinated allele frequency increases in three recently founded lake stickleback populations from Cook Inlet, Alaska (Roberts Kingman et al. 2021; Aguirre et al. 2022). For each of these adaptive regions, we selected the most significant freshwater-adaptive SNP within the region and its neighboring SNPs whose freshwater allele frequencies were highly correlated (*r* ≥ 0.99) across annual time-series data from the three Cook Inlet stickleback populations (Kwakye et al. 2026). There were a total of 33,157 tempo SNPs within 341 freshwater-adaptive regions that met our selection criteria.

The *F_ST_* estimated from tempo SNPs was 0.54 for Rabbit Slough versus Cheney Lake; 0.875 for Rabbit Slough versus Cornelius Lake; and 0.866 for Cheney Lake versus Cornelius Lake. We also estimated the *F_ST_* after filtering out the tempo SNPs, leaving only putatively neutral sites. In this case the *F_ST_* values were similar to those observed across the whole genome; the *F_ST_* between Rabbit Slough and Cheney Lake was 0.032; Rabbit Slough versus Cornelius Lake was 0.083; and Cheney Lake versus Cornelius Lake was 0.072. These results are consistent with results from principal component analyses using genome-wide SNPs (Fig. 1d).

### Comparing Gene Expression from TagSeq and RNAseq

To determine whether our TagSeq approach for measuring gene expression could substantially bias downstream analyses, we compared the gene counts estimated from using TagSeq for gills to those estimated from bulk RNAseq for the same tissue from Verta and Jones (2019) (Methods).

We observed a very high correlation between the gene counts for the marine individuals from Rabbit Slough estimated using the TagSeq pipeline and the marine individuals from the bulk RNAseq from Verta and Jones (2019) (Fig. 2a; *r* = 0.71, *P-value* ≪ 0.001). Similarly, there was a high correlation between the freshwater samples from Verta and Jones (2019) and that from the TagSeq data for both Cheney and Cornelius Lakes (Cheney TagSeq vs. freshwater RNAseq; *r* = 0.705, *P-value* ≪ 0.001; Cornelius TagSeq vs. freshwater RNAseq; *r* = 0.706, *P-value* ≪ 0.001; Fig. 2b and 2c). We observed a weaker correlation when we compared expressed genes from the bulk RNAseq (from the gill tissue) to the expressed genes in the brain from Tagseq (*r* = 0.466, *P-value* ≪ 0.001; Fig. S3). Overall, these results suggest our TaqSeq approach produced biologically meaningful data for downstream analysis with regard to environmental and tissue-specific gene expression variation.

### Differentially Expressed Genes in the Gill and Brain

**Fig. 3.**
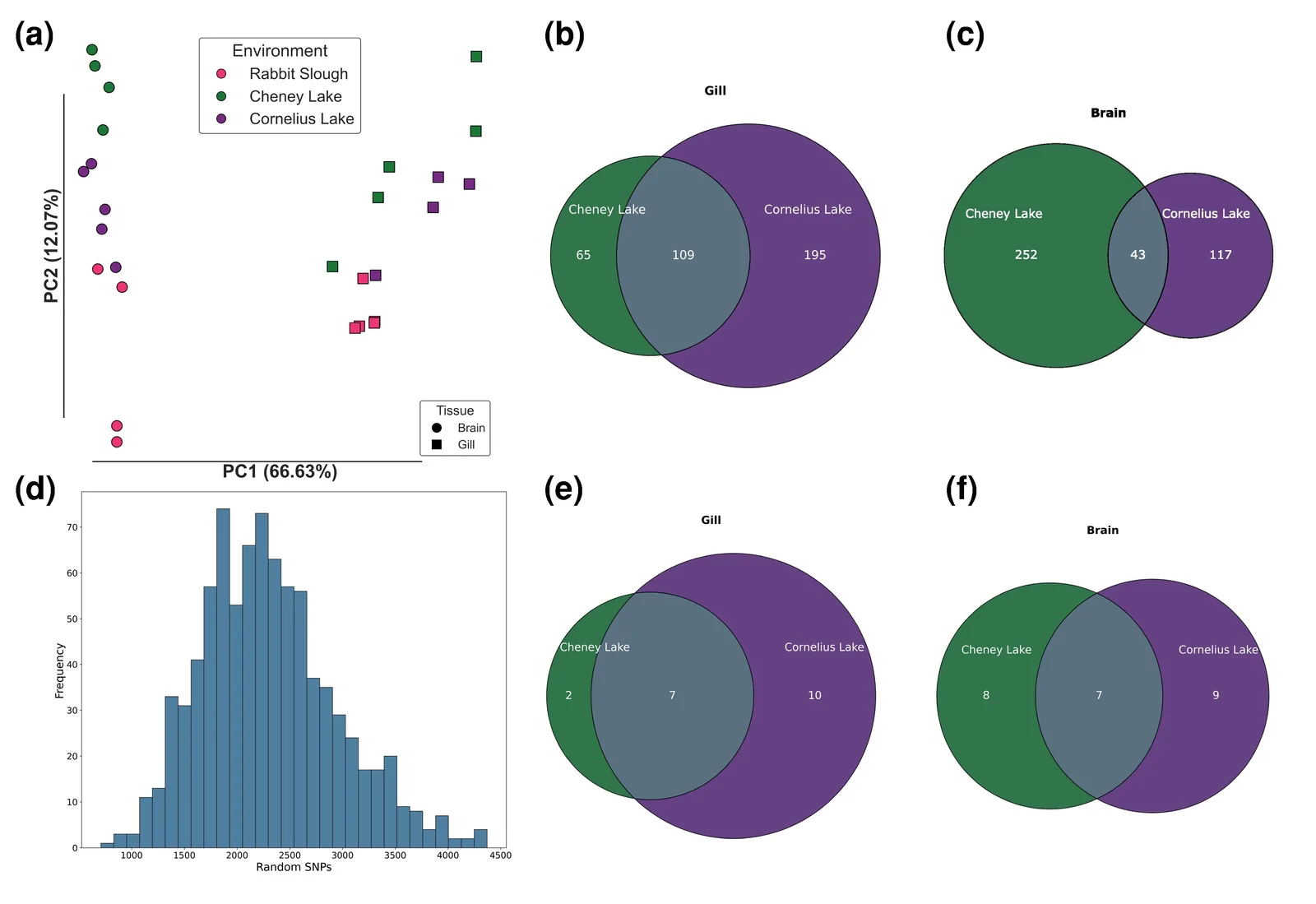
a) Principal components analyses (PCA) using gene expression profiles of all genes across all populations. Venn diagrams of differentially expressed genes (DEGs) when comparing the expression patterns in Cheney Lake and Cornelius Lake samples to Rabbit Slough for b) gill and c) brain. d) An example of our empirical approach for testing for enrichment of F_ST_ outliers and tempo SNPs in DEGs. Venn diagrams of the number of DEGs with F_ST_ outlier SNPs and tempo SNPs for e) gill and f) brain.

Principal component analysis of the normalized gene expression counts showed that the tissue of origin explained the majority of the variation (PC1 = 66.63%) among all the samples (Fig. 3a). After clustering by tissue, samples clustered on PC2 based primarily on population of origin (Rabbit Slough, Cheney Lake, or Cornelius Lake) (Fig. 3a; PC2 = 12.07%).

We detected expression of 14,187 genes in the gill in all samples across all three populations. Following normalization and correction for biological and technical variability using DESeq2 (Love et al. 2014), we identified DEGs between environments, ie Rabbit Slough (marine environment) versus Cheney and Cornelius Lakes (freshwater environments). The comparison between Rabbit Slough and Cheney Lake revealed 174 DEGs with the majority (*n* = 159, 91%) being upregulated in Cheney Lake. There were 304 DEGs between Rabbit Slough and Cornelius Lake, of which 265 (87%) were upregulated in Cornelius Lake. There were 109 DEGs common to the two freshwater environments (expected overlap: 3.65; hypergeometric *P-value*: 8.56e-145, Fig. 3b), 105 (96%) of which were upregulated in both.

The brain had a lower number of total genes (12,290) expressed across the three populations. We found 295 DEGs between Rabbit Slough and Cheney Lake, with 266 (90%) being upregulated in Cheney Lake. There were 123 DEGs found between Rabbit Slough and Cornelius Lake, with 88 (72%) being upregulated in Cornelius Lake (Fig. 3c). Thus, compared with Rabbit Slough samples, Cornelius Lake samples had more DEGs in the gill, while Cheney Lake samples had more DEGs in the brain. There were 40 DEGs that were observed in the brain of both freshwater environments (expected overlap: 2.93; hypergeometric *P-value*: 5.27e-35, Fig. 3c). Among the 40 DEGs found in both freshwater environments, 34 were upregulated in the Cheney Lake samples, while 32 were upregulated in the Cornelius samples. All the six DEGs that were downregulated in the Cheney Lake samples were also downregulated in the Cornelius Lake samples.

We also applied the criteria used in detecting DEGs in our TagSeq dataset to a RNAseq dataset from Verta and Jones (2019) (Methods). There were 192 DEGs, out of which 92 were upregulated in the freshwater samples. Four genes (ENSGACG00000003059, TNNI2, ENSGACG00000009409, and ENSGACG00000008971) were shared between the DEGs from Cheney Lake and the freshwater samples from Verta and Jones (2019) (Fig. S4). However, while all four genes were upregulated in the Cheney Lake population, two of them (ENSGACG00000009409, TNNI2) were downregulated in the freshwater samples from Verta and Jones (2019). Two of the four genes (ENSGACG00000003059 and TNNI2) also overlapped the DEGs in Cornelius Lake (Fig. S5). Despite the limited overlap in the DEGs from Verta and Jones (2019) and our TagSeq dataset, the expression levels of the DEGs from Verta and Jones (2019) were significantly correlated with their expression levels in our TagSeq dataset (r = 0.498, *P*-value = 5.84e-07 for Cheney Lake and r = 0.478, *P*-value = 1.86e-06 for Cornelius Lake; Figs. S6 and S7). This suggests that the lack of observed overlap in DEGs between the two datasets could be explained at least partially by a lack of power, rather than true biological differences.

We next aimed to determine whether the observed systematic upregulation of DEG in our results (but not Verta and Jones (2019)) was a biological artifact of the pre-RNA extraction treatment or a technical artifact introduced by low-coverage RNA sequencing and the bioinformatic pipeline. An alternative biological explanation is that the gene expression measured in the tissues of wild-caught fish may reflect a transient response to environmental conditions in the lakes that triggered a systemic upregulation of the genes. Since the expression of housekeeping genes is expected to remain unchanged across different environments (Thellin et al. 1999; Bustin 2000; Eisenberg and Levanon 2013), we utilized their expression patterns to disentangle the possible cause of the observed upregulation of genes. We determined the expression of housekeeping genes from (Hibbeler et al. 2008), including *actb1*, *gapdh*, *rpl13a*, *hprt1*, *tfb2m*, *taf2*. In our study, the expression of all housekeeping genes remained unchanged in both marine and freshwater environments except *gapdh*, which is known to vary across environmental conditions (Barber et al. 2005; McCurley and Callard 2008; Acharya et al. 2024). *Gapdh* was found to be differentially upregulated in both Rabbit Slough versus Cheney Lake and Rabbit Slough versus Cornelius Lake comparisons (Figs. S8 and S9). We also observed that the expression of these genes remained stable in the marine and freshwater samples reported in Verta and Jones (2019) (Fig. S10) and that the expression levels of these genes were similar in both datasets. Though *gapdh* was not differentially expressed in their dataset, there was an increase in its expression in freshwater individuals (Fig. S10). These results indicate that the systemic upregulation of genes was likely biological, rather than a technical artifact from sequencing and bioinformatic processing.

### Signatures of Positive Selection Associated with DEGs

To determine if DEGs in our dataset were associated with known freshwater-adaptive loci, we examined to what extent the genes overlapped with the 33,157 tempo SNPs. We also analyzed DEGs in relation to loci showing signatures of population differentiation by estimating the per-SNP *F_ST_* between each of the two freshwater populations and Rabbit Slough, considering all SNPs across the genome. For each DEG, we extracted SNPs found up to 10 Kbp upstream of its start site and 10 Kbp downstream of the end of the gene in order to capture putative cis-regulatory regions, including promoters and enhancers. We employed *F_ST_* outlier analyses to select SNPs underlying population differentiation. We set the *F_ST_* outlier threshold to 99th percentile to include only SNPs showing the strongest signals of genetic differentiation, as such SNPs are more likely to be affected by divergent selection rather than demographic processes.

There were 73,679 *F_ST_* outlier SNPs found between Rabbit Slough and Cheney Lake samples, of which 626 SNPs were found in 25 gill DEGs between the same populations (Table S1). Out of these 626 SNPs, 37 of them were tempo SNPs, all of which were associated with the gene *pvalb4* (Table 1). For Rabbit Slough and Cornelius Lake samples, there were 113,081 *F_ST_* outlier SNPs, 2,820 of which were associated with 33 gill DEGs (Table S2). Out of these 2,820 F_ST_ outlier SNPs, 297 were tempo SNPs and were associated with 12 gill DEGs, including *pvalb4* again (Table 1). Out of the 25 gill DEGs associated with *F_ST_* outlier SNPs between Rabbit Slough and Cheney Lake samples, ten of them overlapped the 33 gill DEGs associated with *F_ST_* outlier SNPs between Rabbit Slough and Cornelius Lake samples (Fig. 3e). This overlap was statistically significant (hypergeometric *P-value* = 3.26e-21).

**Table 1:** DEGs with both F_ST_ outliers and tempo SNPs

Gene name	Gene ID	Chromosome	FST outliers	Tempo SNPs
Rabbit Slough versus Cheney Lake gill DEGs				
*pvalb4*	ENSGACG00000012174	chrXI	144	37
Rabbit Slough versus Cornelius Lake gill DEGs				
*hgd*	ENSGACG00000002559	chrXXI	465	8
*nol6*	ENSGACG00000010296	chrXIII	397	10
*si:dkey-205h13.1*	ENSGACG00000011044	chrXII	307	64
*col8a1b*	ENSGACG00000014373	chrI	242	42
*hsc70*	ENSGACG00000007569	chrXX	191	36
*si:dkey-245n4.2*	ENSGACG00000010369	chrXIII	146	10
*sod3b*	ENSGACG00000017840	chrIX	101	30
*pvalb4*	ENSGACG00000012174	chrXI	98	30
*pvalb8*	ENSGACG00000012178	chrXI	92	29
*si:ch211-156l18.7*	ENSGACG00000018299	chrIX	88	14
*tmem168a*	ENSGACG00000018918	chrIV	63	21
*ldha*	ENSGACG00000011270	chrXIX	17	3
Rabbit Slough versus Cheney Lake brain DEGs				
*DOCK2*	ENSGACG00000017885	chrIV	283	41
*ENSGACG00000018361*	ENSGACG00000018361	chrIV	171	29
*dhx58*	ENSGACG00000008740	chrXI	156	80
*n4bp3*	ENSGACG00000018359	chrIV	106	40
*PKP1*	ENSGACG00000009752	chrXII	21	1
Rabbit Slough versus Cornelius Lake brain DEGs				
*arfgef1*	ENSGACG00000002879	chrXXI	890	16
*cmbl*	ENSGACG00000002636	chrXXI	381	7
*gdap1*	ENSGACG00000002727	chrXXI	328	2
*acsl4a*	ENSGACG00000018503	chrIV	311	87
*si:dkey-205h13.1*	ENSGACG00000011044	chrXII	307	64
*PKP1*	ENSGACG00000009752	chrXII	281	5
*ENSGACG00000020069*	ENSGACG00000020069	chrVII	232	109
*ENSGACG00000018030*	ENSGACG00000018030	chrIV	173	28
*ITM2A*	ENSGACG00000018559	chrIV	147	60
*si:dkey-245n4.2*	ENSGACG00000010369	chrXIII	146	10
*ENSGACG00000020330*	ENSGACG00000020330	chrVII	138	84
*strip2*	ENSGACG00000018938	chrIV	104	5
*tmem168a*	ENSGACG00000018918	chrIV	63	21
*dhx58*	ENSGACG00000008740	chrXI	57	35

There were 1,282 *F_ST_* outlier SNPs found between Rabbit Slough and Cheney Lake samples that were associated with 51 of the brain DEGs (Table S3). Of these, 191 were tempo SNPs and were associated with five DEGs (Table 1). There were 4,448 *F_ST_* outlier SNPs found between Rabbit Slough and Cornelius samples that were associated with 29 brain DEGs (Table S4). Of these, 533 were tempo SNPs and were associated with 14 DEGs, including the gene *dhx58*, which was among the five genes also found in the Rabbit Slough and Cheney Lake comparison (Table 1). Out of the 51 brain DEGs harboring *F_ST_* outlier SNPs between Rabbit Slough and Cheney Lake samples, ten of them were among the 29 brain DEGs with *F_ST_* outlier SNPs between Rabbit Slough and Cornelius samples (Fig. 3f, hypergeometric test *P-value* = 2.68e-18).

### Enrichment of Signatures of Selection in DEGs

We next tested whether the *F_ST_* outlier SNPs as well as tempo SNPs were significantly enriched in DEGs compared to the rest of the genome using the following approach. We took the sum of the absolute lengths of each DEG set, which includes all DEGs between the marine and each freshwater environment. We then searched for an equivalent number of genes from the rest of the expressed genes in the respective tissue (either the brain or gill) with similar (± 1Kb) absolute length. We generated 1,000 such random gene sets matching this total length criterion and estimated the number of *F_ST_* outlier SNPs or tempo SNPs within each of them, creating an empirical null distribution for comparison to our observed number of F*_ST_* outlier SNPs or tempo SNPs in each DEG set (Fig. 3d). The tolerance of ± 1Kb accommodates for the likelihood that we cannot get the same number of genes with the exact total length as our query set. If we increase this tolerance distance, we are likely to include more SNPs in our random comparison set, resulting in a higher false negative rate.

We observed that *F_ST_* outlier SNPs were significantly enriched in the DEGs from the comparison between Rabbit Slough and Cornelius Lake samples for the brain tissue (*P-value* = 0.0029). However, we did not find statistically significant enrichment for gill DEGs (*P-value* = 0.267), nor from a comparison between Rabbit Slough and Cheney Lake samples in both tissues (gill *P-value* = 0.495, brain *P-value* = 0.609). Tempo SNPs were significantly reduced in the gill DEGs between Rabbit Slough and Cornelius samples (*P-value* = 0.011). The enrichment of tempo SNPs in brain DEGs between Rabbit Slough and Cornelius samples was not statistically significant (*P-value* = 0.233). For the comparison between the Rabbit Slough and Cheney Lake samples, the tempo SNPs were not significantly enriched in either the brain (*P-value* = 0.872) or the gill (*P-value* = 0.73).

### Parvalbumin Expression in the Gill

**Fig. 4.**
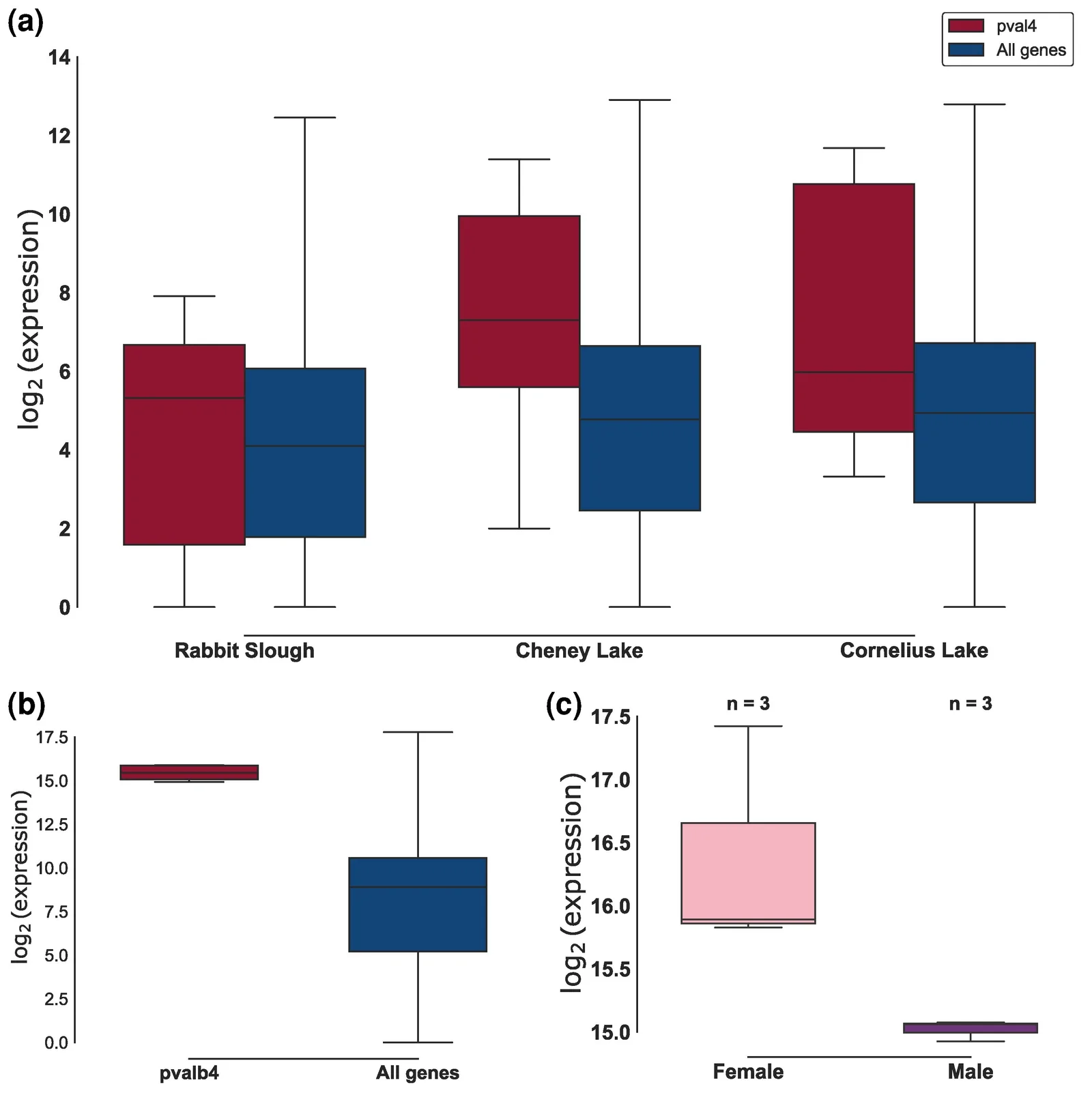
a) Expression of parvalbumin (*pvalb4)* compared to all other genes in the gill of Rabbit Slough, Cheney, and Cornelius Lakes. b) Expression of *pvalb4* gene in the gill in samples from Verta and Jones (2019) in comparison with other expression levels of other genes in the genome. c) Expression of *pvalb4* gene in the gill samples from Verta and Jones (2019) between females (*n* = 3) and males (*n* = 3).

Considering that the *pvalb4* gene was differentially upregulated in the gill in both freshwater environments (Fig. 4a) as well as harboring substantial *F_ST_* outlier SNPs and tempo SNPs, we explored its expression in the gill RNAseq dataset from Verta and Jones (2019). Based on the criteria we used in identifying DEGs in the TagSeq dataset, *pvalb4* was not significantly differentially expressed between the marine and freshwater samples from (log2FC = −0.107, adjusted *P-value* = 0.980). However, when we considered the expression of this gene compared to the rest of the genes in the genome, *pvalb4* was among the most highly expressed genes in both marine and freshwater samples (Fig. 4b), suggesting that it may be involved in one or more key functions in threespine stickleback. The gene also showed elevated expression in females compared to males in this dataset (Fig. 4c), although this trend was marginally nonsignificant (Mann–Whitney U = 9.0, *P-value* = 0.05) and not replicated in our TagSeq dataset.

### Genetic Load in DEGs

We next aimed to determine the predicted effects of freshwater-/marine-divergent SNPs associated with DEGs on protein function and structure. In this analysis, we focused on tempo SNPs that were also *F_ST_* outliers for all sets of DEGs and referred to these SNPs as “candidate SNPs.” In total, there were 946 candidate SNPs across all four DEG sets. We annotated candidate SNPs based on their predicted effects on protein function using snpEff (Cingolani et al. 2012; Janssen et al. 2023), which assigns SNPs to impact classes: low, moderate, high, and modifier. We also determined the extent to which candidate SNPs are conserved over evolutionary time, which may be indicative of their functional importance, using their site-specific Genomic Evolutionary Rate Profiling (GERP) scores (Cooper et al. 2010; Davydov et al. 2010) (Methods). SnpEff can assign multiple effects to the same variant. For example, if a gene has multiple transcripts, the program assigns effects to all transcripts; or if a variant is located upstream of one gene and downstream of another, it reports effect of the variant on both genes. We therefore report all effects assigned to each SNP.

**Fig. 5.**
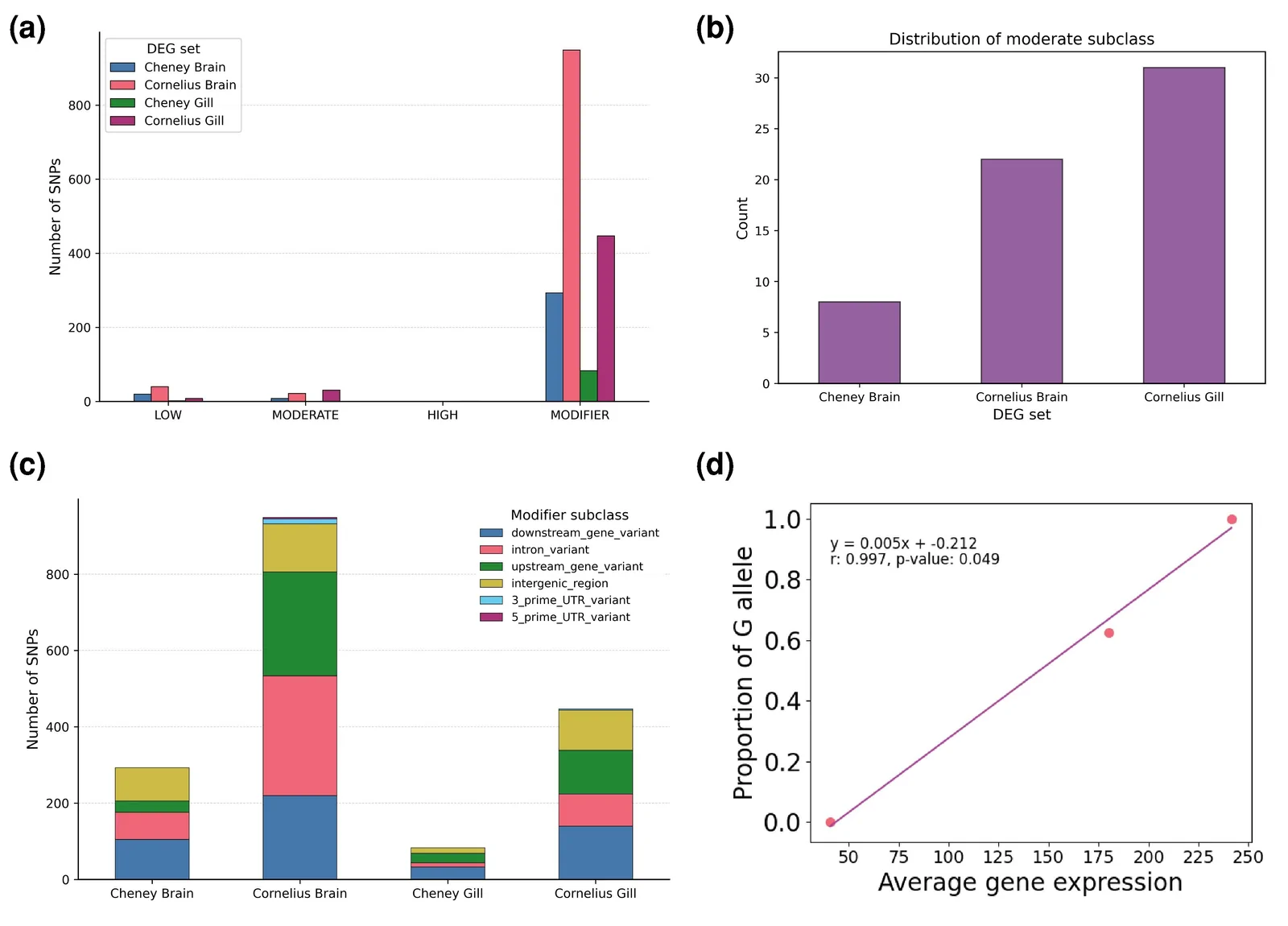
Predicted molecular effects of candidate SNPs. a) Distribution of molecular effects of 946 candidate SNPs as predicted by snpEff. Breakdown of the various components that make up the b) moderate impact class (all of which were missense variants). c) Modifier impact class. d) Correlation between the average gene expression in the gill tissue and the proportion of G alleles at a SNP (chrI:26106268 G > T) predicted to have a high impact on the gene *col8a1b*.

We observed that most candidate SNPs had either low, moderate, or modifier effects, with the majority being modifiers (Fig. 5a). The modifier subclass was predominantly used to annotate upstream or downstream variants, consistent with previous suggestions that freshwater adaptation is mostly mediated by cis-regulatory changes (Jones et al. 2012; Verta and Jones 2019; Mack et al. 2023) (Fig. 5b). The moderate effect variants were predominantly missense variants: 8 missense variants in Rabbit Slough versus Cheney Lake brain DEGs, 22 in Rabbit Slough versus Cornelius Lake brain DEGs, and 31 in Rabbit Slough versus Cornelius Lake gill DEGs (Fig. 5c). The eight missense variants in the Rabbit Slough versus Cheney Lake brain DEGs were distributed across three DEGs, with *dhx58* harboring three of them. The 22 missense variants in the Rabbit Slough versus Cornelius Lake brain DEGs were found across eight DEGs, including five in the gene *acsl4a*, five in the gene *gpr174*, and one in *dhx58* that was also identified in the Rabbit Slough versus Cheney Lake brain DEGs. There were 13 of the missense variants from the Rabbit Slough versus Cornelius Lake gill DEGs that were found in the gene *col8a1b*, while three were found in the gene *sod3b* and two in the gene *DHX15* (Table S5).

We identified a candidate SNP in the Rabbit Slough versus Cornelius Lake gill DEGs on chromosome I (chrI:26106268 G > T) with a putatively high impact on the molecular function and/or structure of the *col8a1b* gene product. This SNP potentially leads to a premature stop codon c.1838C > A (p.Ser613*). The expression of *col8a1b* in the gill was substantially correlated with the allele counts in the three populations (Fig. 5d, *r* = 0.98, *P* = 0.122) but not in the brain (Fig. S11, *r* = −0.031, *P* = 0.980). All five individuals in the Cornelius sample were homozygous for the G allele, while the 20 Rabbit Slough individuals were homozygous for the T allele, with the observed heterozygosity in the Cheney Lake population being 0.65 (expected heterozygosity = 0.30). The SNP is also found upstream of the gene *jagn1b* and is predicted to be in the modifier class for this gene; however, this gene was not expressed in either tissue.

Out of the 946 candidate SNPs, we retrieved the GERP scores for 139 of them. Out of these, 115 (83%) had GERP scores above 0. However, the distribution of GERP scores in sticklebacks is right-skewed, with a median (50th percentile) value of 7.55—90th, 95th, and 99th percentiles of 16, 17.20, and 19.90, respectively (Fig. S12). Out of the 139 candidate SNPs with GERP scores, 68 were found in the 50th percentile, 18 in the 90th percentile, five in the 95th percentile, and 0 in the 99th percentile. Out of the five in the 95th percentile, four were found in Rabbit Slough and Cornelius brain DEGs (all of which were associated with the gene ENSGACG00000020069) and one in a Rabbit Slough and Cornelius gill DEG, which was associated with the *hgd* gene. All the SNPs had one allele fixed in the Rabbit Slough sample, while the alternate allele was fixed in the Cornelius sample.

We also determined the evolutionary constraints on specific DEGs harboring substantial *F_ST_* outlier SNPs and tempo SNPs such as *pvalb4* and missense variants such as *dhx58* and *col8a1b*. For sites where we retrieved the GERP scores in *pvalb4* and *dhx58*, we observed that all of the GERP scores were below the genome-wide median (Figs. S13 and S14). There were no sites with GERP scores in *col8a1b*.

### Gene Ontology

To identify molecular processes that are enriched in each set of DEGs, we performed gene ontology (GO) analyses. After adjusting for false positive rate, GO terms such as muscle contraction and extracellular matrix organization were enriched in the DEGs from Rabbit Slough versus Cheney Lake and Rabbit Slough versus Cornelius Lake. There were no enriched GO terms in brain DEGs in both environments (Kolberg et al. 2023).

## Discussion

In this study, we leveraged the repeated, rapid adaptation observed in threespine stickleback to explore whether genomic signatures of selection might influence gene expression changes during adaptation to freshwater environments for two important tissues, gill and brain. The stickleback population in one of the freshwater environments, Cheney Lake, was recently founded using stickleback sampled from Rabbit Slough, where we sampled the marine individuals. The population in the other freshwater environment, Cornelius Lake, likely diverged from anadromous ancestors much further in the past. However, our analyses indicated a substantial convergent freshwater adaptation that has occurred at both lakes. We identified common signatures of selection associated with DEGs for samples collected from stickleback populations inhabiting both of these independent freshwater environments for both the brain and gill tissues. The SNPs were mostly located in regulatory regions of genes, consistent with previous studies (Jones et al. 2012; Verta and Jones 2019).

### Signatures of Selection Are Predominantly Found in Regulatory Regions

We found a SNP with putatively high impact on protein structure and function of *col8a1b* gene which codes for the alpha-1 chain of the collagen VIII protein. A transcript of the gene *col8a1b* gene is found on the reverse strand of chromosome I and has three exons, but the start codon is found on exon 2 which means that the first exon is primarily located in the 5′ UTR. The SNP results in a stop codon at c.1838C > A in *col8a1b*, truncating the 746-amino-acid protein at position 613. This stop mutation occurs in the third and last exon of *col8a1b*, and thus the transcript likely does not undergo nonsense mediated mRNA decay. Instead, it likely produces a truncated protein that may be dysfunctional because collagen VIII assembly requires the truncated carboxyl-terminus domain to initiate trimerization (Boudko et al. 2009). The stop codon may be acting as a cis-eQTL, influencing *col8a1b* expression in a dosage-dependent manner; the T (A on the reverse strand) allele is associated with significantly reduced transcript levels in the gill, a pattern consistent with incomplete dominance. The SNP deviated substantially from Hardy–Weinberg equilibrium, which could indicate substantial selection pressure. It is possible that this gene is important during development in threespine stickleback as it is involved in vertebral column segmentation during development in zebrafish (Gray et al. 2014).

Aside from this SNP, we observed that the majority of the SNPs showing signatures of selection and associated with DEGs were located in cis*-*regulatory regions and included variants up- and downstream of genes, as well as synonymous variants, consistent with previous studies (Jones et al. 2012; Verta and Jones 2019). There were also missense variants in genes such as *dhx58*, *acsl4a*, *gpr174*, *col8a1b*, *sod3b*, and *DHX15*. *DHX15* is an RNA helicase that can function as a viral sensor in the innate immune system (Pattabhi et al. 2019; Xing et al. 2021) and may be required for antiviral immunoregulation during freshwater adaptation. *Sod3b* is involved in the regulation of oxidative stress during adaptation to extreme conditions in an Antarctic blackfin icefish (Kim et al. 2019).

### Genes Involved in Molecular Processes in the Gill and Brain

We focused our study on the gill and brain based on their key roles in osmoregulation and behavior during transitions from marine to freshwater environments (Bonga 1973; Bell and Sih 2007; Judd 2012; Hasan et al. 2017). In the gill, we found genes such as *tnnt1*, *pvalb4*, and *atp6v1e1a* to be differentially expressed in both freshwater environments, relative to their anadromous ancestor, and also enriched for signatures of selection. *Tnnt1* encodes a subunit of troponin that binds to tropomyosin (Pato et al. 1981; Wei and Jin 2016). Through its role in anchoring the troponin complex to the thin filament, *Tnnt1* contributes to calcium-dependent regulation of gill muscle contraction, which may facilitate sustained ventilation and gaseous exchange across the gill filaments. Our GO analysis showed that the term muscle contraction was enriched in DEGs in the gill of samples collected from both freshwater environments. Although muscle contraction depends on intracellular calcium release rather than directly on environmental calcium, the reduced availability of external calcium in freshwater may impose selective pressures on calcium homeostasis (Pinto et al. 2010; Schredelseker et al. 2010). Such pressures could indirectly favor changes in the regulation of calcium-sensitive contractile proteins, including components of the troponin complex, in gill-associated muscles.

In addition to *tnnt1*, the gene atp6v1e1a was differentially expressed in the gills. This gene encodes for a subunit of an ATP-dependent intracellular protein pump, which potentially is involved in influx of calcium into the gill. Although *cacnb1* was found to be differentially expressed only in Cheney Lake, it encodes for a subunit of voltage-gated calcium channel that was found to be differentially expressed during a salinity change (Taugbøl et al. 2022).

The gene *pvalb4*, which encodes the parvalbumin protein that chelates calcium, thereby contributing to calcium regulation (Heizmann 1984), was differentially upregulated in both freshwater environments and enriched with a large number of *F_ST_* outliers and tempo SNPs. Interestingly, ENSGACG00000009409, which is unannotated in the stickleback genome, was among the only two genes that were upregulated in both our dataset and that of Verta and Jones (2019). This gene is also an ortholog of *pvalb4* of multiple species, including *Poecilia reticulata* (guppy) and *Neolamprologus brichardi* (fairy cichlid). Parvalbumin has been implicated in marine–freshwater divergence in at least one other study, which found it to be downregulated in the gill of freshwater stickleback (Verta and Jones 2019). Our reanalysis of a subset of the dataset from Verta and Jones (2019) also indicated that the gene is highly expressed in sticklebacks in both marine and freshwater environments. Parvalbumin has been shown to be involved in fast muscle contraction (Celio and Heizmann 1982), which may explain why it is highly expressed in a fish species with fast swimming activities (Zhang et al. 2017). In individuals of the *Xenopus allofraseri* that exhibit high levels of locomotion, *pvalb4* was found to be differentially expressed compared to those with reduced mobility (Ducret et al. 2021). Parvalbumin has also been linked to sustained locomotion in fishes (Seebacher and Walter 2012). These findings suggest that *pvalb4* may play key roles during the adaptation of threespine stickleback to freshwater environments, possibly by allowing the fish to escape predators. However, we note that the effect of an increase in *pvalb4* expression in anti-predation may be well tested with gene expression experiments that include other tissues, particularly the muscle tissue.

During transition from marine to freshwater habitats, genes involved in cellular ion exchange may be targets of selection (Divino et al. 2016; Velotta et al. 2022) and important for many recent freshwater invasions (Lee and Bell 1999). In threespine stickleback, the comparatively lower calcium concentration in freshwater environments has often been suggested as a selective force driving lateral plate polymorphisms between the contrasting environments (Giles 1983; Smith et al. 2014) [but see (Reimchen 1983; MacColl and Aucott 2014)]. Our results suggest that the contrasting calcium concentrations between environments may be driving phenotypic changes in the gill, perhaps more so than in the lateral plates. The enrichment of both tempo SNPs and *F_ST_* outliers in DEGs such as *pvalb4* in both a recently established freshwater population and a long established population may indicate that calcium concentration has long been a selective force driving freshwater adaptation for a long time.

In the brain, we found genes such as *strip2*, *dhx58*, and *acsl4a* were found to be enriched with signatures of selection. *Acsl4a* encodes for a ligase that converts long-chain fatty acids into fatty acyl-CoA esters. In freshwater environments, the diet of threespine stickleback is deficient in long-chain unsaturated fatty acids such as docosahexaenoic acid (DHA), which are abundant in marine environments. Freshwater populations therefore have to rely on cellular biosynthesis of these fatty acids for normal growth and development. *Acsl4a* was previously found to be involved in lipogenesis (Lopes-Marques et al. 2013) in cavefish, suggesting that it may play key roles in fatty acid metabolism in stickleback. Kitano et al. (2019) identified genes that showed parallel expression divergence whereby genes were repeatedly up- or downregulated in brain samples collected from both Canadian and Japanese streams compared to marine samples. Among the genes showing parallel expression divergence from Kitano et al. (2019), only the gene ENSGACG00000011044 (*si:dkey-205h13.1*) was found to be differentially expressed in our brain samples (but also in our gill samples). This gene encodes for a transmembrane protein and is relatively conserved across many vertebrates.

In sticklebacks, Ishikawa et al. (2019) showed that *fads2*, a gene involved in DHA biosynthesis, is crucial for the colonization of new freshwater environments although mechanisms involving other DHA biosynthesis genes may be required (Twining et al. 2026). *Fads2* is the rate-limiting enzyme in the polyunsaturated fatty acid biosynthesis pathway, and *acsl4a* acts downstream of this enzyme. It remains unclear how the polyunsaturated fatty acid biosynthesis pathway is implicated in the brain and the specific selective pressure involved, but it is possible that this pathway may be involved in neurogenesis, synaptic function, or inflammation (Bazinet and Layé 2014).

Inflammation in the brain may also explain the differential expression of *dhx58*, a gene that encodes an ATP-dependent RNA helicase that regulates type I interferon involved in antiviral innate immunity (Quicke et al. 2019). In threespine stickleback, it is not only parasites such as the cestode, *Schistocephalus solidus*, that is ubiquitous in the organism's freshwater habitats, but the fish may coevolve with viruses such as *Picornaviridae*, a family of nonenveloped RNA viruses (Hahn and Dheilly 2019). The presence of viruses may explain why genes involved in antiviral immunoregulation such as *dhx58* are differentially expressed. In two Alaskan populations with varying degrees of infection, Wohlleben et al. (2024) found *dhx58* expression to be associated with *S. solidus* infection, although type I interferon which is regulated by the RNA helicase *dhx58* is involved in antiviral immunity.

### Comparison with Previous Studies

We found a strong correlation when comparing the gene counts estimated from TagSeq with bulk RNAseq from Verta and Jones (2019). We also observed a stable expression of housekeeping genes in both environments in our dataset and the RNAseq from Verta and Jones (2019), except for *gapdh*, which was differentially expressed in freshwater in our dataset. This differential expression of *gapdh* in our dataset likely reflects environmental stress encountered by wild-caught fish, consistent with previous studies that highlight the gene's high expression variance in response to oxidative stressors (Barber et al. 2005; Camacho-Jiménez et al. 2023; Babington et al. 2025). Although *gapdh* was not differentially expressed, its expression level was increased in the freshwater samples compared with the marine samples from Verta and Jones (2019). This was likely due to a reduction in the variation of the expression levels of the gene as a result of the common garden treatment of the fish prior to the measurement of the gene expressions.

Though correlated overall, the number of expressed genes we identified in the gill (∼14,500) was slightly less than 16,688 genes from Rodríguez-Ramírez and Peichel (2025). In the brain, there was also a limited overlap between the DEGs from Kitano et al. (2019) and our study. These differences in expressed genes between these studies and our own likely result from the different sequencing approaches. While traditional RNAseq used in Rodríguez-Ramírez and Peichel (2025) sequences full length of transcripts, TagSeq (in our study) involves sequencing only the 3′ end of mRNAs, which may impact the ability to detect the expression of certain genes, particularly those with longer transcripts (Crow et al. 2022). Traditional RNAseq also leads to the detection of more DEGs, compared to approaches that sequence the 3′ end of mRNAs such as TagSeq (Ma et al. 2019). Microarray based studies can also lead to differences in estimated gene expression compared with sequencing based approaches such as TagSeq in our study (Ordas et al. 2011).

### Upregulation of Genes During Freshwater Adaptation

Our analyses showed that the vast majority of the genes that were differentially expressed in both tissues were upregulated in the freshwater environments. A previous study that monitored the expression of immune specific genes, including genes characteristic of innate immunity, immunosuppression, and helper T lymphocytes, in stickleback from wild populations and lab-raised fish showed that wild fish differed in the expression of immune specific genes characteristic of innate immunity, immunosuppression, and helper T lymphocytes (except the Th1-subtype of helper T cells) compared to lab-raised fish (Robertson et al. 2016). Importantly, wild fish had increased expression of all the immune specific genes. The differences in the patterns of gene expression in immune specific genes between wild-caught fish and lab-raised fish indicate that possibly the difference in the number of DEGs between this study and the previous studies was due to treatment of the samples prior to RNA extraction. In the studies that Rodríguez-Ramírez and Peichel (2025) reanalyzed, the fishes were captured from the wild and reared in the lab for a period before extracting RNA, which, by design, depletes some variation. In the current study, we used RNA from tissues collected directly from wild-caught fish, which may be more variable, reducing power to detect genes with only subtle expression changes.

In Gibbons et al. (2017), the authors subjected marine and freshwater stickleback fish to varying salinities and measured the patterns of differential gene expression in the gill tissue. When they compared gene expression in the gill of fish reared in 0 ppt and 30 ppt, they observed almost equal proportions of upregulated genes to downregulated genes. They observed similar patterns when they compared marine and freshwater ecotypes (Gibbons et al. 2017). Verta and Jones (2019) also treated marine and freshwater ecotypes to varying degrees of salinities and measured the differential expression of genes in the gill. Our reanalysis of a subset of this dataset also indicated that the number of up- and downregulated genes were almost equal. It may be that studies that measured gene expression using threespine stickleback raised in common garden environments eliminate certain triggers—such as pathogens or predators in the natural setting that cause certain genes to turn on. On the other hand, quantifying gene expression from wild-caught fish might capture expression patterns induced by environmental variables that turn on expression of genes. For instance, *gapdh* may be upregulated in our wild-caught freshwater individuals due to an increased demand for ATP. In addition, Verta and Jones (2019) suggested that DEGs found in regions of marine–freshwater divergence tended to be predominantly upregulated, which is in line with our finding that most of the DEGs were upregulated.

Our findings suggest that threespine stickleback in freshwater environments may mount a systematic upregulation of genes associated with critical physiological processes, such as calcium regulation and fatty acid metabolism. Our analysis revealed that *gapdh*, a metabolic gene that is frequently used as a housekeeping gene, was differentially expressed in the freshwater populations. This shift in expression may reflect the elevated metabolic costs of active ion regulation required to maintain homeostasis in ion-poor environments. Thus, increased metabolic and physiological demands appear to drive a broad transcriptomic shift toward increased expression of essential survival genes in freshwater habitats. Possibly, DNA hypermethylation silences the expression of these genes in the marine environment, and the change in environment to freshwater induces genome-wide hypomethylation (Hu and Barrett 2023), which then increases the expression of genes by making them more accessible for transcription. Genome-wide hypomethylation is however inconsistent with studies that identified hypermethylation in both marine and freshwater samples (Smith et al. 2015). It is also possible for chromatin remodeling and accessibility to mediate such widespread upregulation of genes when marine stickleback encounter a new freshwater environment.

## Future Directions

The SNPs associated with DEGs that are both tempo SNPs and F*_ST_* outliers could be important targets for future functional analyses. Further studies are also needed to validate our observation of gene upregulation in freshwater adaptation. Although we subjected all the specimens to the same downstream processing to ensure maximum similarity in the conditions we could control, such as the amount of time elapsed from collecting the specimen from the lake and harvesting the organs, future studies that utilize larger sample sizes could clarify the effect of confounding variables in gene expression patterns measured from wild-caught fish.

## Conclusion

Recent advancements in genomic scans have revealed many signatures of selection. Yet it remains unclear how these signatures of selection mediate phenotypic changes. In this study, we identified single nucleotide polymorphisms (SNPs) associated with DEGs that may underlie freshwater adaptation. These SNPs were predominantly found in regulatory regions of the genes and likely have small effects on protein function and structure. A SNP with predicted high impact on protein function and structure predominantly showed dosage-dependent effect on the gene expression. Together, the results add to growing efforts to connect alleles identified by more than two decades of genome scans to the phenotypes they might influence.

## Materials and Methods

### Sampling of Threespine Stickleback, RNA Preservation, and Extraction

Threespine sticklebacks were collected between June 2 and June 10, 2023, from Cheney Lake (61.204, −149.76), Cornelius Lake (61.628, −149.255), and Rabbit Slough (61.532, −149.266) in Alaska, USA. Minnow traps were deployed to collect stickleback samples from these three locations. Five traps were set at Cheney Lake on the west arm of the Northern peninsula of the lake at 4:45 PM on June 2, 2023 (air temperature 10.5 °C, water temperature 12 °C), yielding 30 sticklebacks upon recovery on June 3 at 2:32 PM. On June 3, ten traps were deployed at Rabbit Slough at the stream flow near the Glenn Highway at 12:00 PM (air temperature 18 °C, water temperature 13 °C). We recovered the traps on June 4 at 12:20 PM, yielding 431 fish. Ten traps were also set at Cornelius Lake, near North Engstrom Road, at 1:30 PM on June 3 (air temperature: 16.5 °C; water temperature: 12.5 °C) and recovered on June 4 at 11:00 AM, yielding 375 fish. For all the three locations, a subset of captured individuals (total 13 for Cheney Lake, 41 for Rabbit Slough, and 47 for Cornelius Lake) were kept. We sacrificed five to ten individuals shortly after collection using MS-222 and then harvested the brain and gill tissues. The tissues were immediately preserved in RNAlater and ultimately stored at −80 °C for subsequent molecular analysis. All procedures were approved by the College of New Jersey Institutional Care and Use of Animals Committee (protocols 1908 to 001MW1A1 and 2002 to 001MW1A3).

### Measurement of Standard Lengths

For each fish from which we harvested brain and gill tissues, we took a photo and estimated the standard length (SL) using ImageJ software from the photos. The SL is measured as the distance from the anterior tip of the upper jaw (premaxilla) to the posterior end of the last vertebra (hypural plate).

### RNA Extraction and Sequencing

We removed frozen tissues from −80 °C and allowed them to thaw gradually by first putting them in a refrigerator for approximately 30 min before exposing them to room temperature. For each tissue, we cut about 3.5 to 4.5 mg for extraction, and the rest of the tissue was stored in a −80 °C freezer. We used the Qiagen RNAeasy kit (RNeasy Mini Kit (50), Cat no.74104) to extract the RNA from tissues by following the manufacturer instructions with slight modifications. We added 2-Mercaptoethanol (1ML 2-Mercaptoethanol, Cat No: 97622, Sigma Aldrich) to the lysis buffer. After extraction, we quantified the RNA concentration in each sample using the Qubit Fluorometer 3.0 set to the High Sensitivity option for RNA. The concentrations of the RNA were normalized within the range required by the Genomic Sequencing and Analysis Facility of the University of Texas at Austin. The minimum concentration of the 30 specimens was 1.7 ng/ul, while the maximum was 82.7 ng/ul. The samples were sent to the Genomic Sequencing and Analysis Facility of University of Texas at Austin for library preparation and RNA sequencing on the Illumina NovaSeq 6000 SR100.

### TagSeq

We sequenced the extracted RNA using a revised version of the TagSeq approach (Lohman et al. 2016). TagSeq involves sequencing only the 3′ end of mRNAs, which reduces the cost compared to whole mRNA sequencing. This approach increases the power of an experimental design as more samples can be sequenced at relatively low cost. However, this increased power comes at the expense of reduced information. In particular, TagSeq is unable to distinguish alternatively spliced variants from one genomic locus, as well as polymorphisms within the gene locus. Nevertheless, this approach fits the goal of this study, which is to leverage existing genetic variation information for various populations of threespine stickleback, including from Rabbit Slough and Cheney Lake, two of the populations used in this study.

### Bioinformatic Processing

We adapted the pipeline for processing TagSeq raw reads from https://github.com/z0on/tag-based_RNAseq. We concatenated read files belonging to the same specimen and tissue (each specimen had brain and gill tissues) and trimmed adapters using the software cutadapt v1.14 (Martin 2011) with Python 2.7.15 and command line parameters:—-a AAAAAAAA -a AGATCGG -q 15 -m 25. We then aligned the reads to the stickleback reference genome stickleback_v5 (https://stickleback.genetics.uga.edu/downloadData/v5_assembly/stickleback_v5_assembly.fa.gz) (Peichel et al. 2020) using bowtie2 with the –local option. We used the script samcount.pl (https://github.com/z0on/tag-based_RNAseq/blob/master/samcount.pl) to count isoforms mapping to each gene.

### Processing of Bulk RNAseq

In order to compare our newly generated TagSeq data to existing, comparable bulk RNAseq, we reprocessed a subset of the Verta and Jones 2019 (Verta and Jones 2019) dataset from the gill, including three marine and three freshwater individuals collected from Little Campbell River, British Columbia, Canada. The marine samples were collected from 49.016, −122.780, while the freshwater individuals were collected from 49.012, −122.625. There were one male and two female marine individuals and two females and one male freshwater individuals. We downloaded the reads from SRA (accession numbers: SRR8868247, SRR8868248, SRR8868249, SRR8868250, SRR8868251, SRR8868252, SRR8868254). Subsequently, we performed adapter removal using trim-galore. We then mapped the reads to the stickleback reference genome stickleback_v5 (https://stickleback.genetics.uga.edu/downloadData/v5_assembly/stickleback_v5_assembly.fa.gz) (Peichel et al. 2020) and counted the number of reads per gene using STAR (Dobin et al. 2013). Similar to Verta and Jones (2019), we used the following settings in STAR aligner: –readFilesCommand zcat –outSAMtype BAM SortedByCoordinate –quantMode GeneCounts –outFilterIntronMotifs RemoveNoncanonicalUnannotated –chimSegmentMin 50 –alignSJDBoverhangMin 1 –alignIntronMin 20 –alignIntronMax 200000 –alignMatesGapMax 200000 –limitSjdbInsertNsj 2000000.

### Differential Gene Expression

All samples had more than one million reads, with the exception of one brain sample from Rabbit Slough. We therefore removed this sample from all downstream analyses. We used DESeq2 (Love et al. 2014) to perform normalization on the count data before performing differential gene expression. DESeq2 models count data using a negative binomial distribution and apply median-of-ratios normalization (Anders and Huber 2010). To reduce noise from lowly expressed transcripts and improve statistical power in downstream analyses, we removed all genes with base mean count of less than ten across all samples. We performed differential gene expression analysis between marine and freshwater populations separately for the brain and gill tissues. Gene expression counts were modeled as *count ∼ environment + sex*, using a negative binomial generalized linear model implemented in DESeq2. We defined DEGs as genes with log2FC > 2 and adjusted *P-value* < 0.05.

### Genomic Datasets and Processing

In order to contextualize RNA expression with patterns of genetic variation, we utilized genomic data from a 2009 Rabbit Slough sample previously published by Roberts Kingman et al. 2021 (Roberts Kingman et al. 2021). We trimmed adapters with AdapterRemoval (ver. 2.2.2) (Lindgreen 2012) and then mapped reads to the Threespine Stickleback genome version gasAcu1-4 (Peichel et al. 2017; Roberts Kingman et al. 2021) using bwa mem -M (v0.7.15-r1140) (Li and Durbin 2009). We added read groups using Picard before being merged with samtools (Danecek et al. 2021). We also marked duplicates using Picardtools. We performed base recalibration using BaseRecalibrator from GATK ver3.7 (McKenna et al. 2010; Van Der Auwera et al. 2013).

### Population Structure and Signatures of Selection

Due to the relatively lower mean coverage of the Cornelius Lake individuals (mean coverage of approximately 6× compared to the approximately 20× coverage for Rabbit Slough and 30× for Cheney Lake), we performed pairwise *F_ST_* using angsd (Korneliussen et al. 2014), which is designed for low-coverage genomic data. We first estimated the site allele frequency likelihood for each population using angsd (using 20 individuals from Rabbit Slough, 20 individuals from Cheney Lake, and 5 individuals from Cornelius Lake). We then calculated the 2d site frequency spectrum (SFS) between each population pair (Rabbit Slough vs. Cheney Lake, Rabbit Slough vs. Cornelius Lake, and Cheney Lake vs. Cornelius Lake) using realSFS. The pairwise F_ST_ was then estimated using the site allele frequency likelihood and the 2d sfs. We also performed PCA using a combination of angsd and pcangsd (Meisner and Albrechtsen 2018). Again, we used angsd to estimate the genotype likelihoods, but using all individuals in the three populations combined (*n* = 45). We then used pcangsd to estimate the covariance matrix and the eigen function in *R* to compute the eigenvalues and eigenvectors.

### Enrichment of Signatures of Selection in Differentially Expressed Genes

We used bedtools intersect to find the SNPs located in DEGs. To capture putative cis-regulatory regions, including promoters and enhancers, we included SNPs found within 10Kbp up- and downstream of the gene. The distance between enhancers of genes and the promoters they interact with in eukaryotic organisms can vary from a few Kbp to 1Mbp, with certain enhancers found in other genes (Birnbaum et al. 2012; Ritter et al. 2012). While a 10Kbp threshold does not capture long range enhancers, this threshold serves as a balance between gene specific SNPs and those that can overlap multiple other genes. We determined the enrichment of F_ST_ outliers (we defined F_ST_ outliers as values greater than 99th percentile; see below) in DEGs compared to the rest of the genome using Fisher's exact test.

### Genetic Load

We annotated the multi-population VCF using snpEff [version SnpEff 4.3t (build 2017 to 2011-24 10:18)] (Cingolani et al. 2012). We built a custom database for the stickleback genome gascu 1 to 4 (Roberts Kingman et al. 2021) using the following command: java -jar snpEff.jar build -gtf22 -v Gascu_1-4 -noCheckCds -noCheckProtein. We then ran snpEff annotation with and without the -noShiftHgvs option. As we mapped the RNA-seq data to the v5 of stickleback genome, we lifted over the bed files of all DEGs from the v5 to v4 using liftOver and chain files from https://stickleback.genetics.uga.edu/downloadData/chain_files/v5_to_v4.chain.txt. We also determined genetic load across the DEGs using GERP scores. For this analysis, we downloaded conservation scores (https://ftp.ensembl.org/pub/release-112/compara/conservation_scores/65_fish.gerp_conservation_score/) for the 65 fish multiple alignment (https://ftp.ensembl.org/pub/release-112/emf/ensembl-compara/multiple_alignments/65_fish.epo_extended/) from the Ensembl release 112. We then lifted over these sites from version 5 (https://ftp.ensembl.org/pub/release-112/fasta/gasterosteus_aculeatus/dna/Gasterosteus_aculeatus.GAculeatus_UGA_version5.dna_rm.toplevel.fa.gz) to version gascu 1 to 4 using liftOver and chain files from https://stickleback.genetics.uga.edu/downloadData/chain_files/v5_to_v4.chain.txt.

### Gene Ontology

We performed gene set enrichment analyses using GOrilla (Eden et al. 2009, 2007), which allows specification of target and background gene lists. We chose the Danio rerio (zebrafish) database since there is no option for threespine stickleback in GOrilla. We used biomaRt (Durinck et al. 2009) in R to download zebrafish orthologs of stickleback genes. For DEGs with orthologs in the zebrafish genome, we ran the gene enrichment analysis in GOrilla using the following options: two unranked lists of genes as the running mode, all as GO option and *P*-value threshold of 0.001.

## Supplementary Material

evag222_Supplementary_Data

## Data Availability

All sequenced raw reads have been deposited on Sequence Read Archive (www.ncbi.nlm.nih.gov/sra) under accession code PRJNA1427159.
