# Supplementary material for: Genomic Signatures of Selection Are Enriched in Differentially Expressed Genes in Sticklebacks Adapting to Contrasting Environments
Source: Genome Biol Evol. 2026 Aug 30;18(9):evag222. doi: 10.1093/gbe/evag222 (PMC13583502; doi:10.1093/gbe/evag222)
Supplement: Supplementary file 1 [file evag222_supplementary_data.pdf]

Supplementary Figures

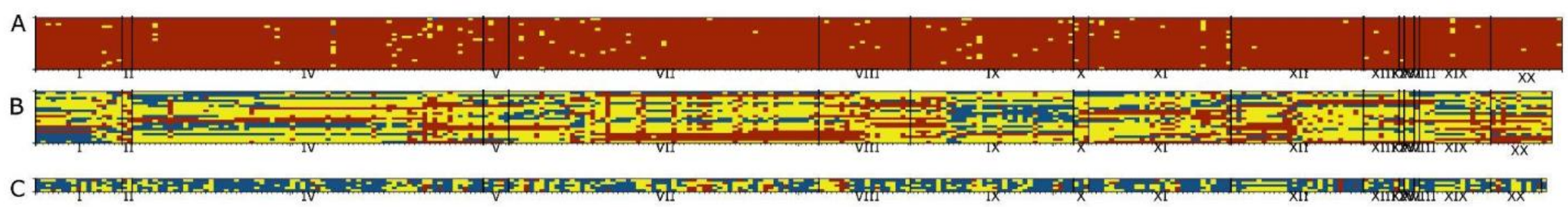

Fig. S1 Genotype at regions of the genome identified by (Roberts Kingman et al. 2021) to be underlying rapid freshwater adaptation for **A)** Rabbit Slough **B)** Cheney Lake **C)** Cornelius Lake samples.

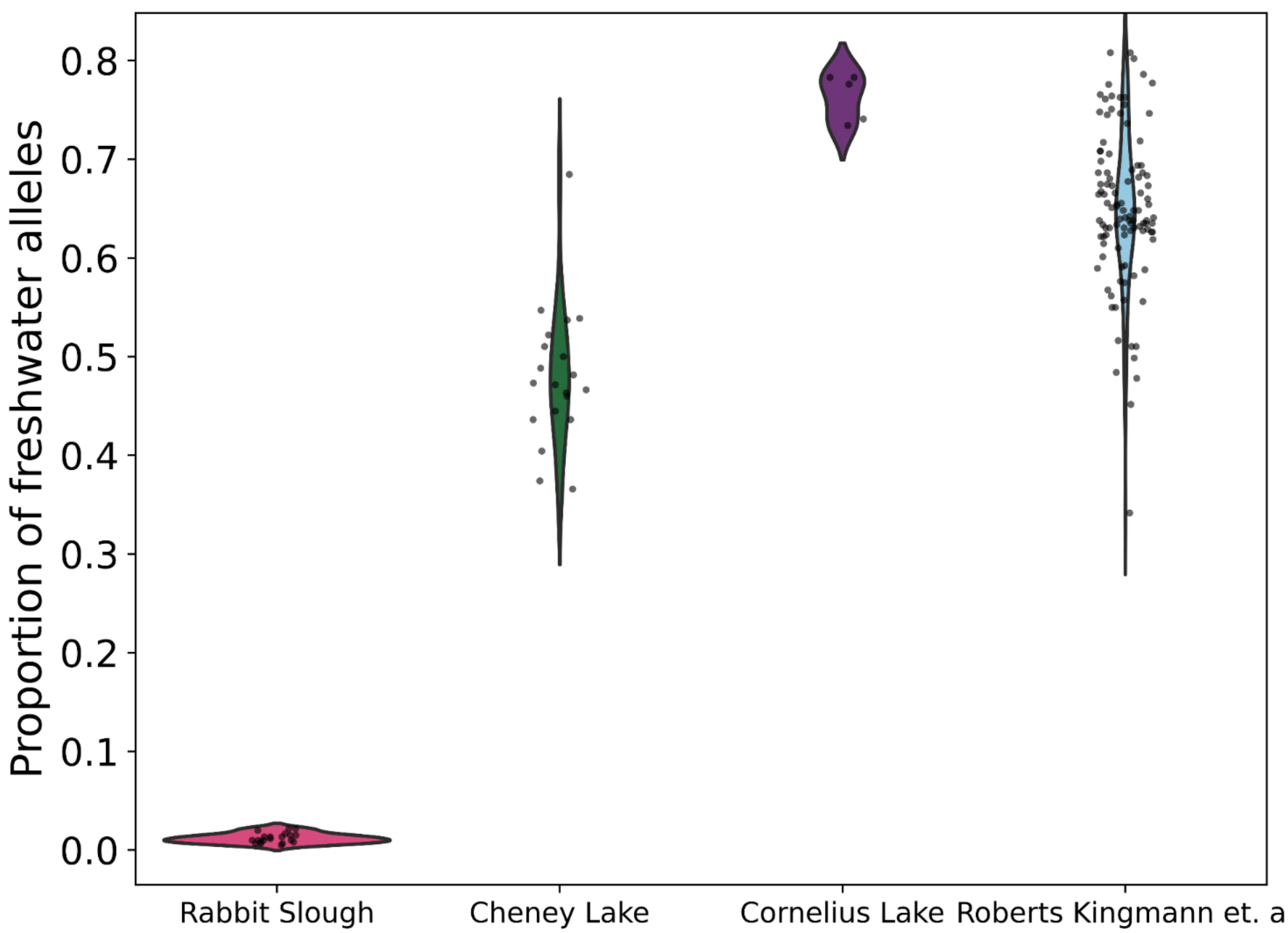

Fig. S2: Proportion of freshwater adaptive alleles for individuals collected from Rabbit Slough, Cheney Lake, Cornelius Lake, and genomes from freshwater populations published in Roberts Kingmann et al. (Roberts Kingman et al. 2021)

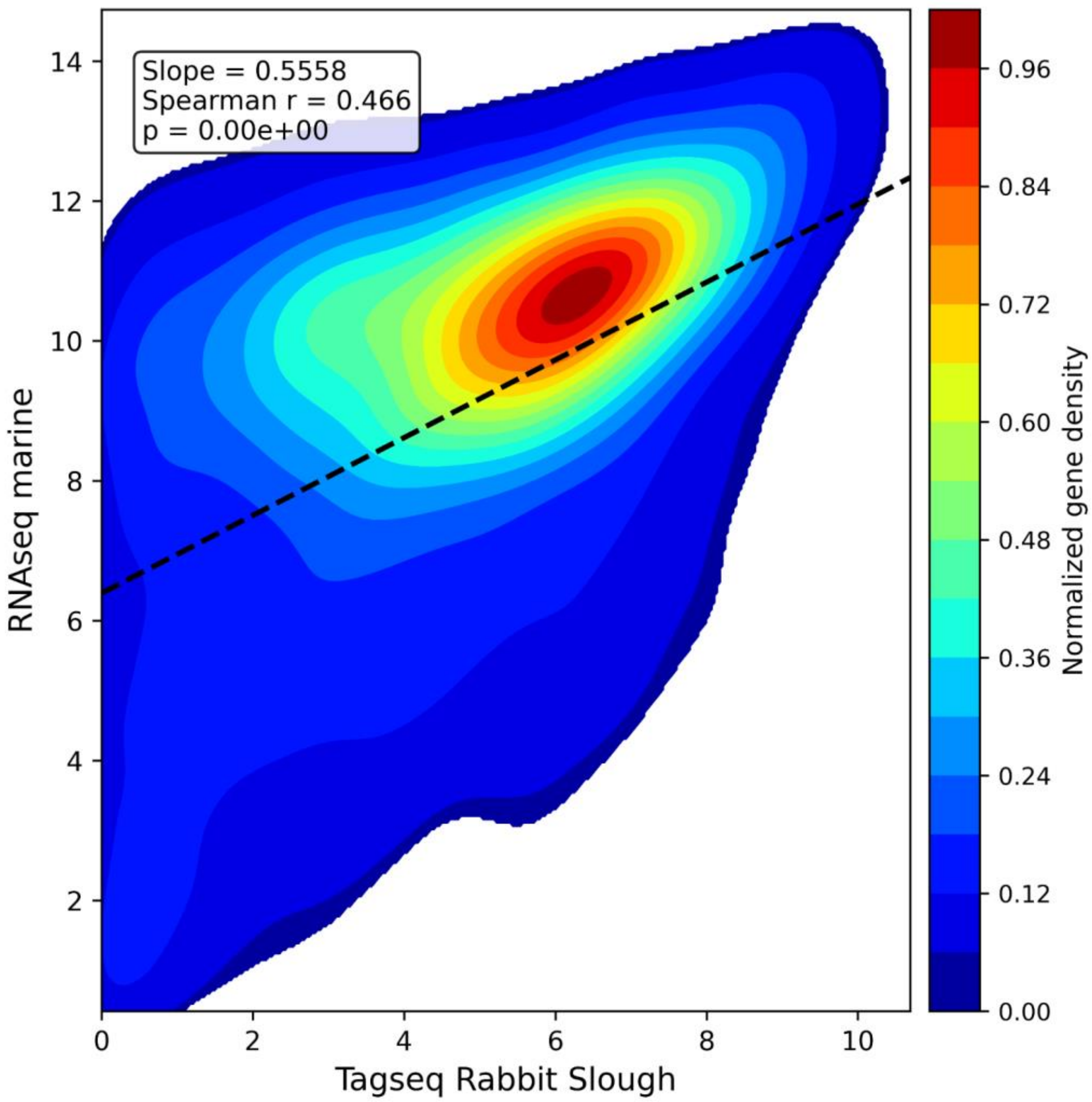

Fig. S3 Correlation of gene counts estimated using TagSeq for genes expressed in the brain for Rabbit Slough individuals vs genes expressed in the gill for marine individuals collected from Little Campbell River

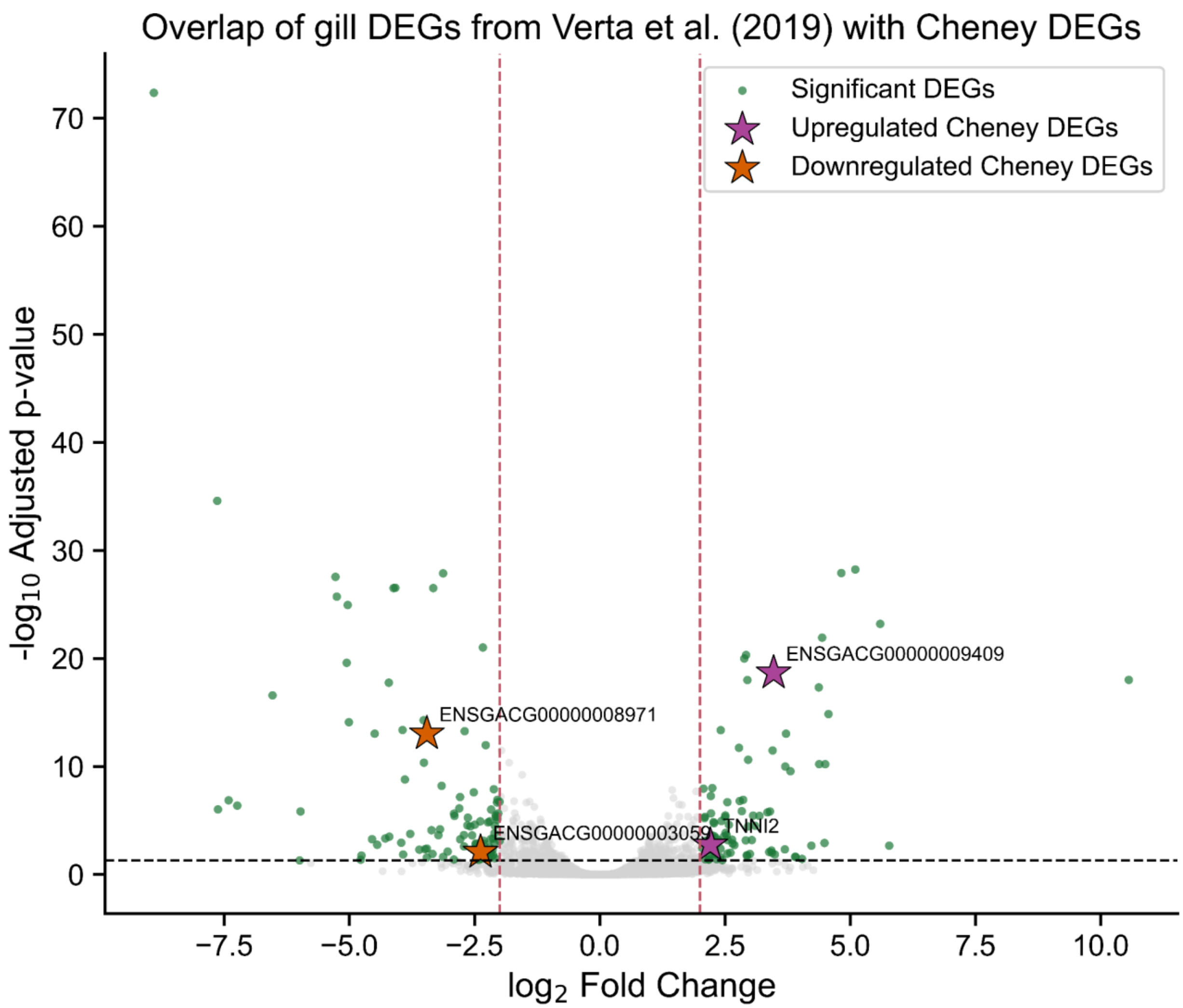

Fig. S4:. Volcano plot of the genes from Rabbit Slough vs Cheney Lake with the DEGs from Verta and Jones (2019) overlaid onto it. Most DEGs from Verta and Jones (2019) have low fold changes in the TagSeq dataset. For the genes present in the two datasets, we have labeled those that were differentially expressed in the same direction in both datasets.

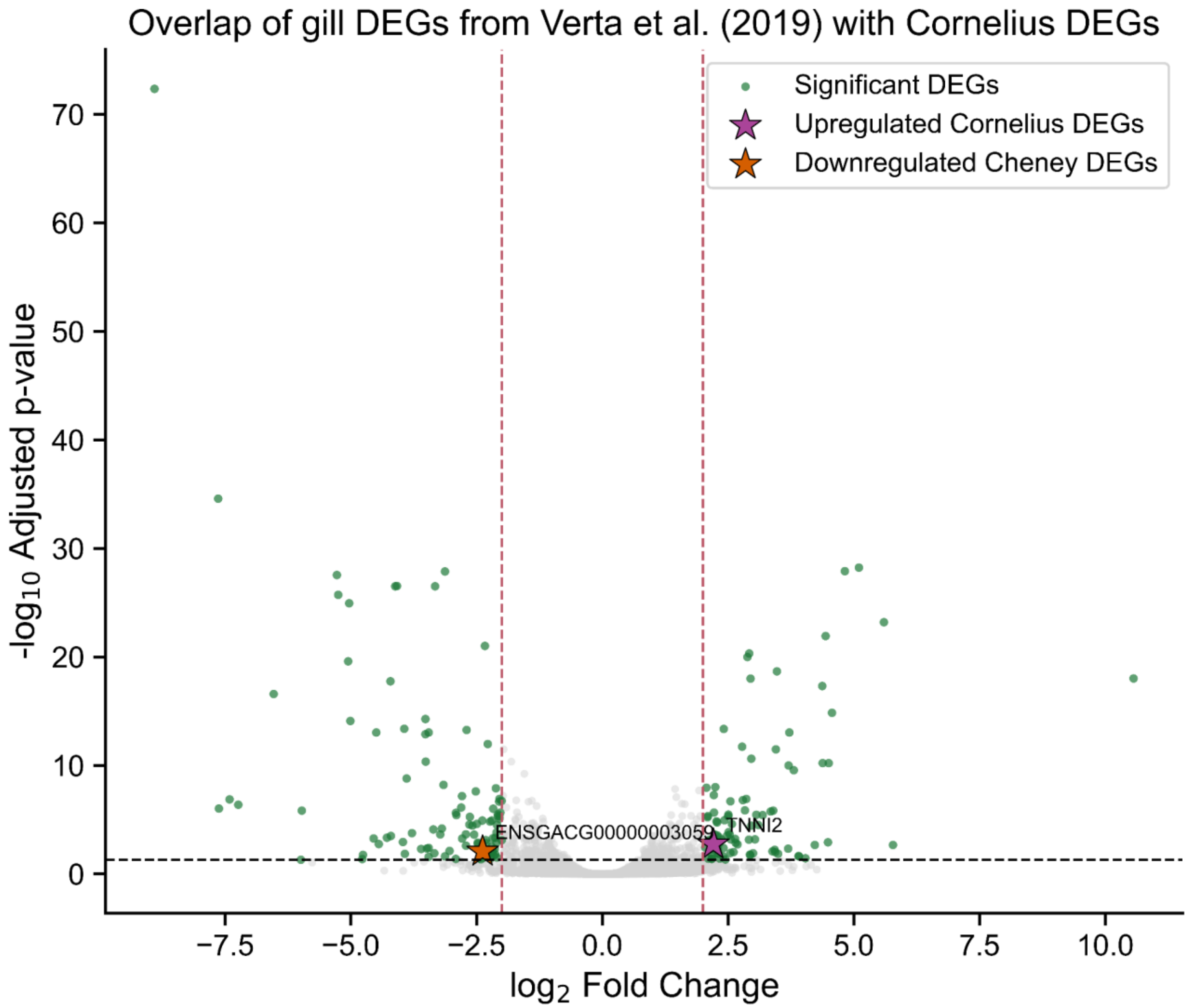

Fig. S5: Volcano plot of the genes from Rabbit Slough vs Cornelius Lake with the DEGs from Verta and Jones (2019) overlaid onto it. Most DEGs from Verta and Jones (2019) have low fold changes in the TagSeq dataset. For the genes present in the two datasets, we have labeled those that were differentially expressed in the same direction in both datasets.

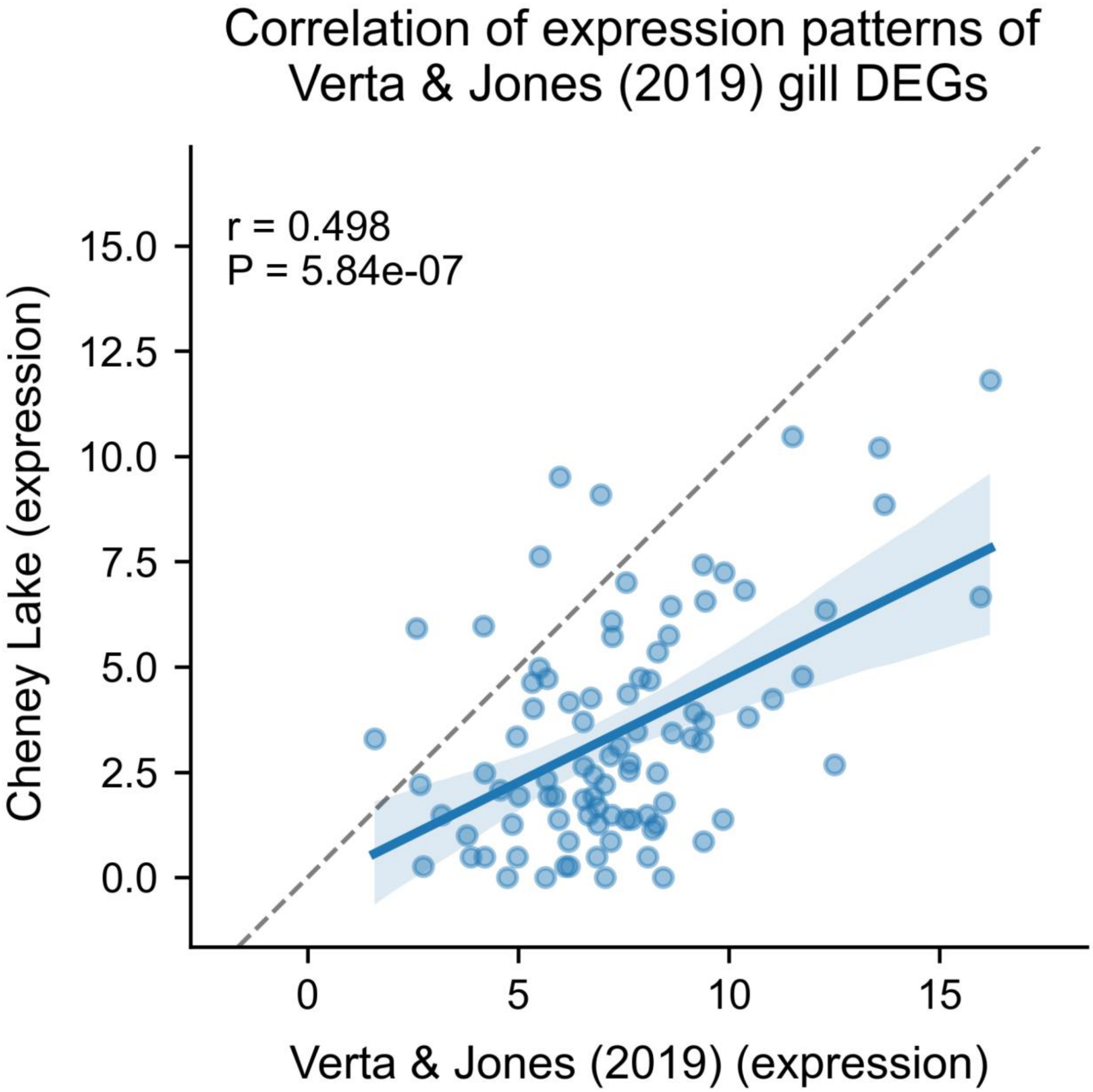

Fig. S6: Correlation of expression patterns of DEGs identified from Verta and Jones (2019) and their expression levels in Cheney Lake gill samples

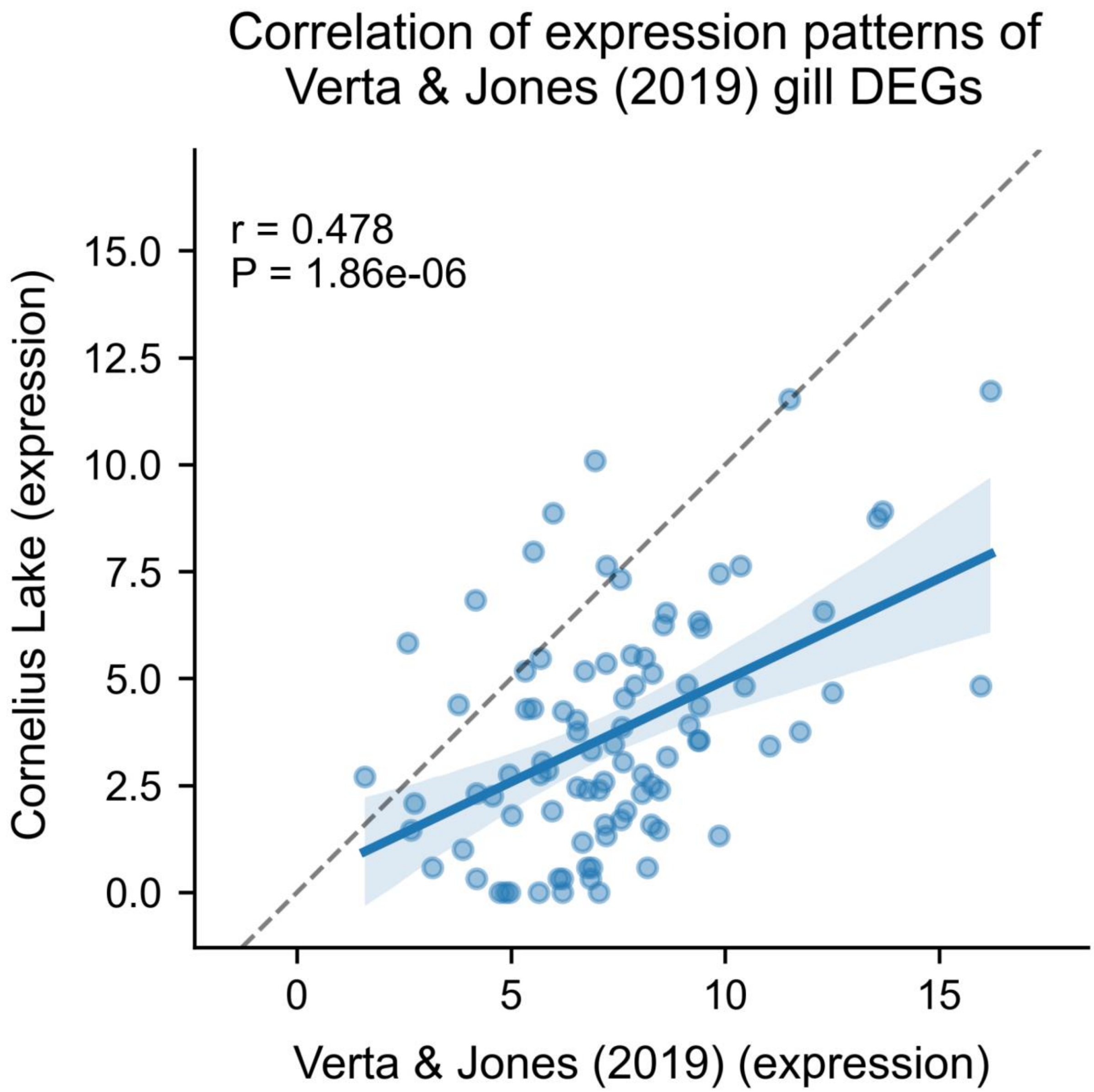

Fig. S7: Correlation of expression patterns of DEGs identified from Verta and Jones (2019) and their expression levels in Cornelius Lake gill samples

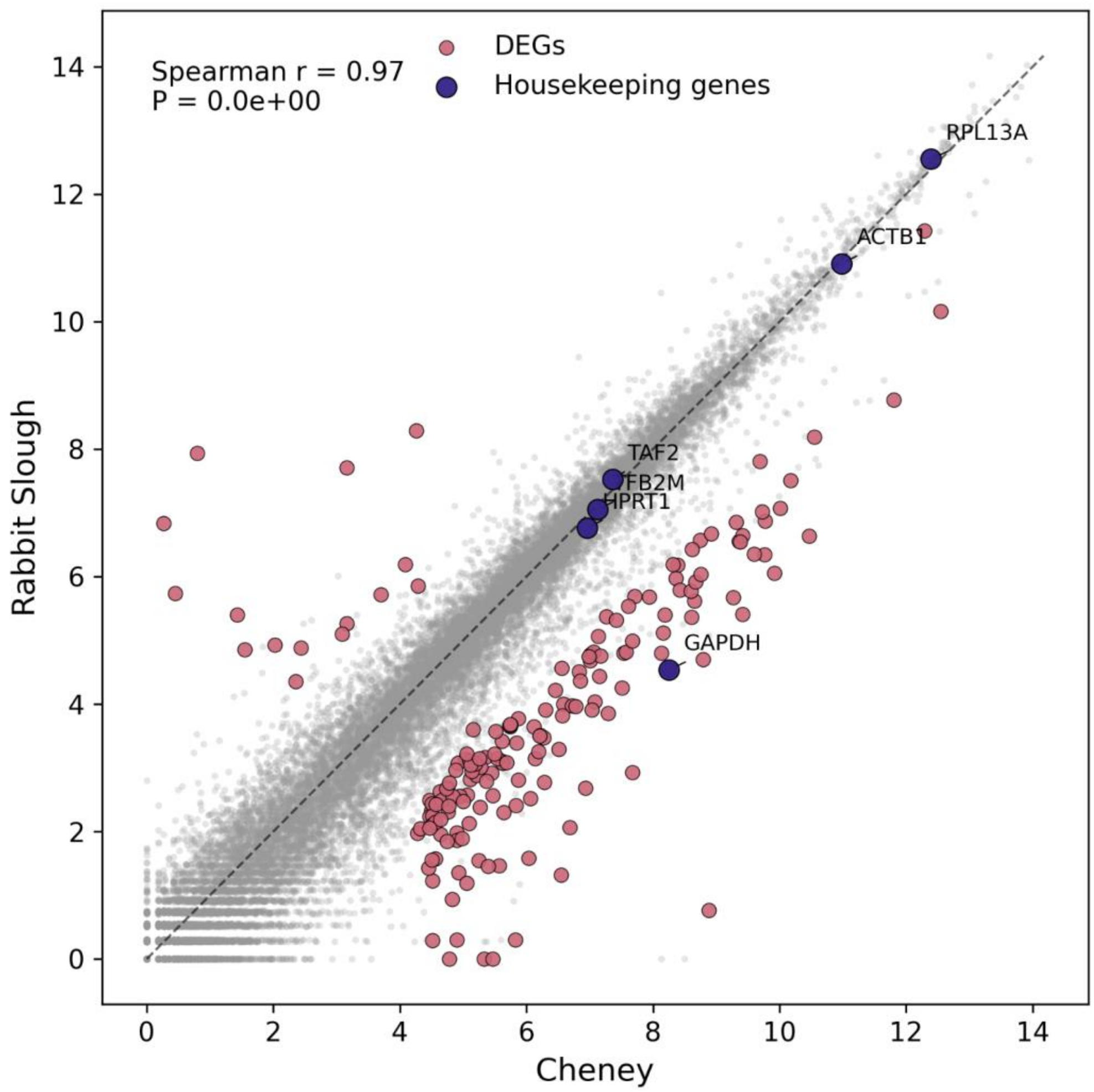

Fig. S8: Correlation of gene expression between marine and freshwater stickleback populations. Correlation analysis of normalized transcript counts in gill tissue between Cheney and Rabbit Slough populations. Each point represents an individual gene. Differentially expressed genes (DEGs) are highlighted in red and housekeeping genes in blue.

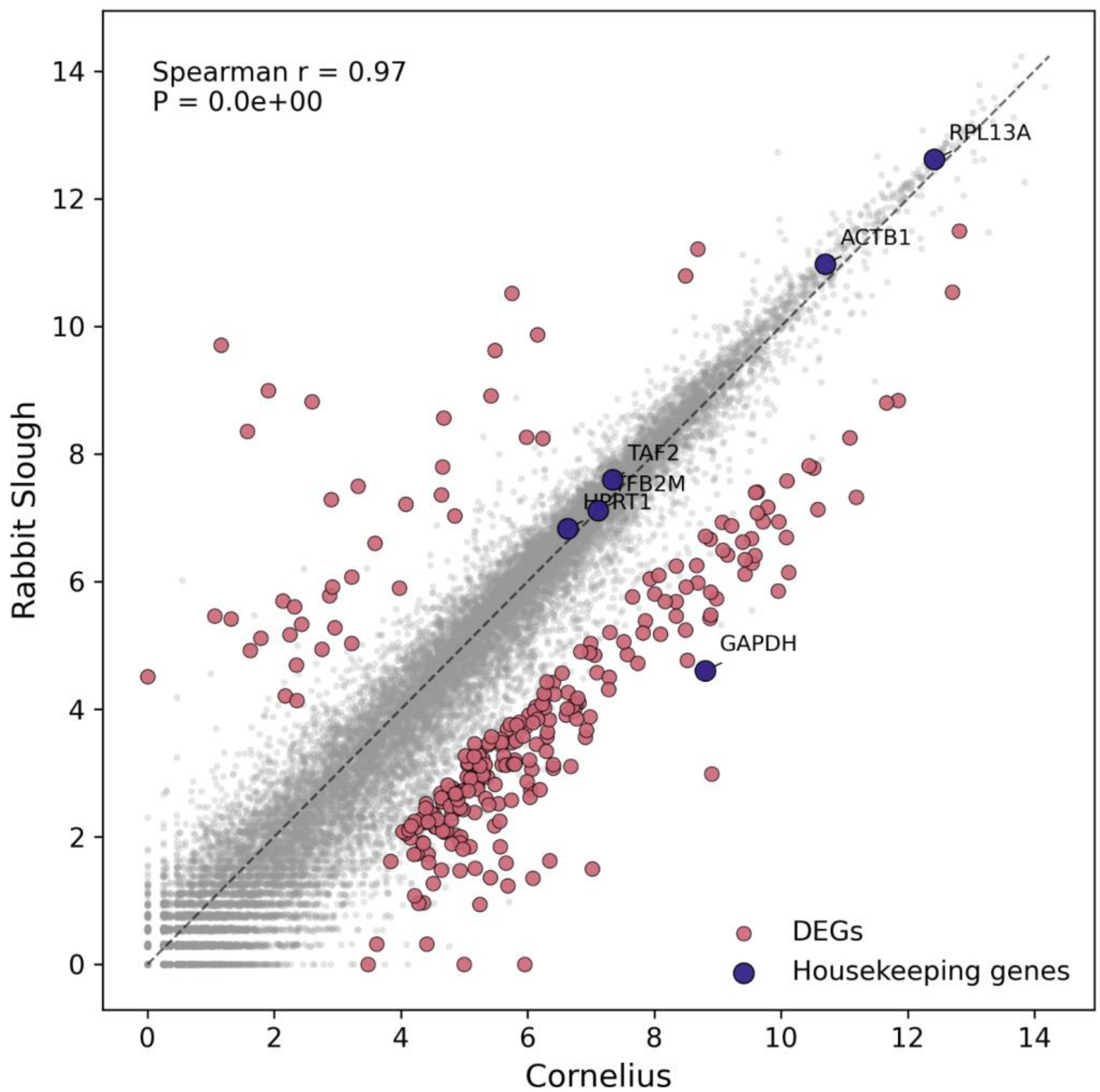

Fig. S9: Correlation of gene expression between marine and freshwater stickleback populations. Correlation analysis of normalized transcript counts in gill tissue between Cornelius and Rabbit Slough populations. Each point represents an individual gene. Differentially expressed genes (DEGs) are highlighted in red and housekeeping genes in blue.

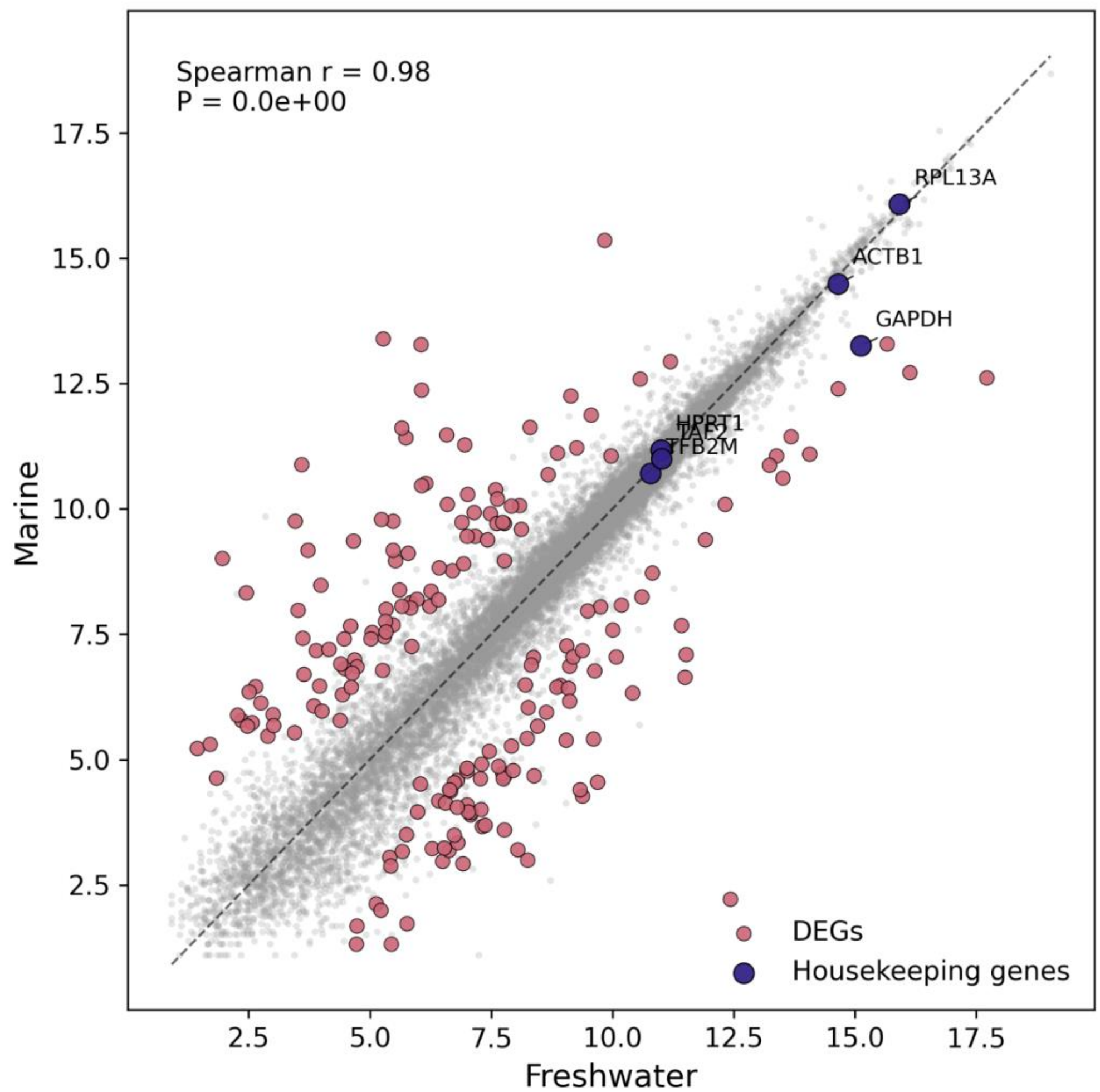

Fig. S10: Correlation of gene expression between marine and freshwater stickleback populations. Correlation analysis of normalized transcript counts in gill tissue between marine and freshwater populations from Verta and Jones 2019. Each point represents an individual gene. Differentially expressed genes (DEGs) are highlighted in red and housekeeping genes in blue.

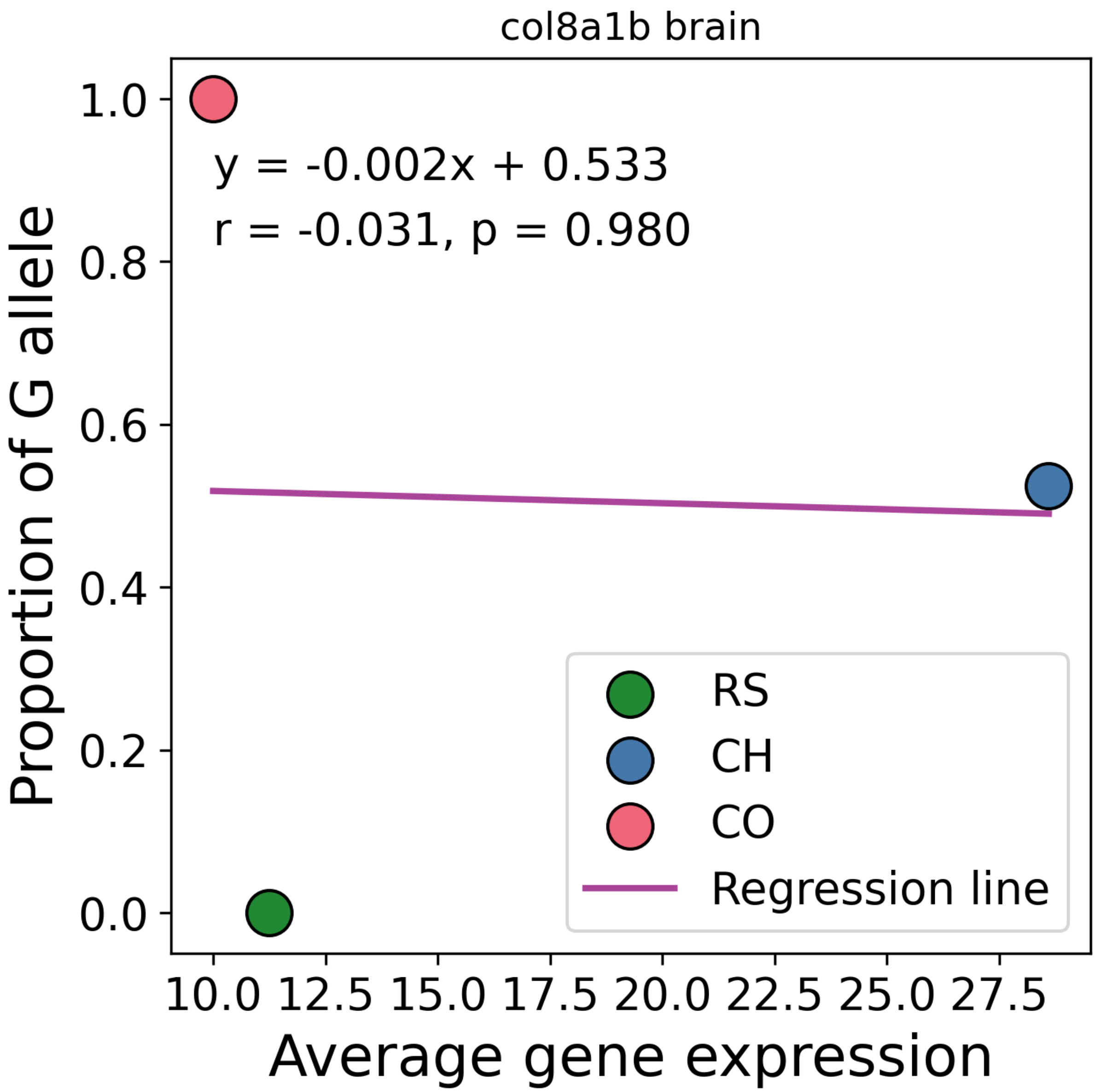

Fig. S11: Correlation between the average gene expression in the brain tissue and the proportion of G alleles at a SNP((chrI:26106268 G > T) predicted to have a high impact on the gene *col8a1b*.

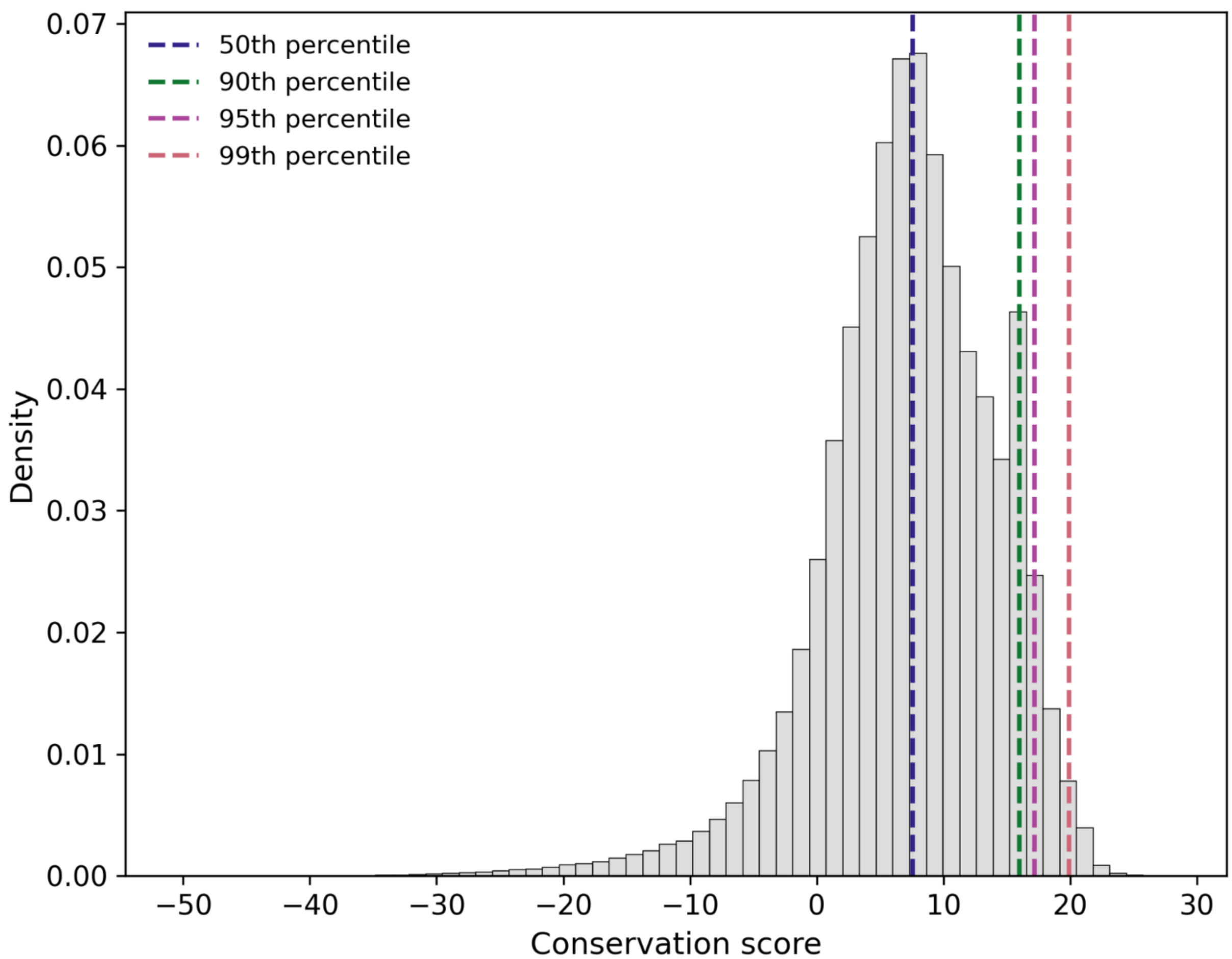

Fig. S12: Genome-wide distribution of GERP scores for threespine stickleback. We downloaded the pre-computed GERP scores for the 65 fish multiple alignment from ensembl (https://ftp.ensembl.org/pub/release-112/emf/ensembl-compara/multiple_alignments/65_fish.epo_extended/).

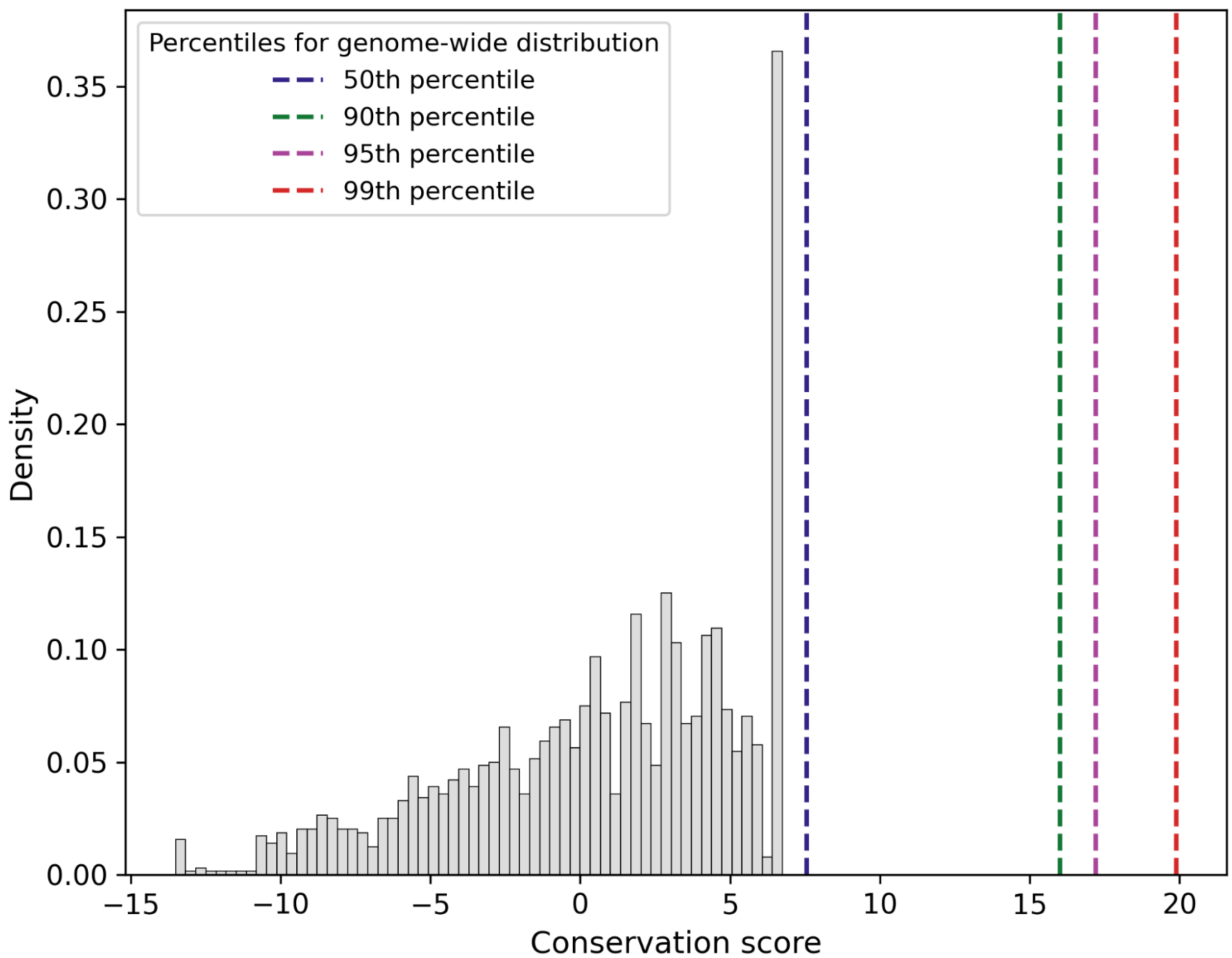

Fig. S13: Distribution of GERP scores for the gene *dhx58*

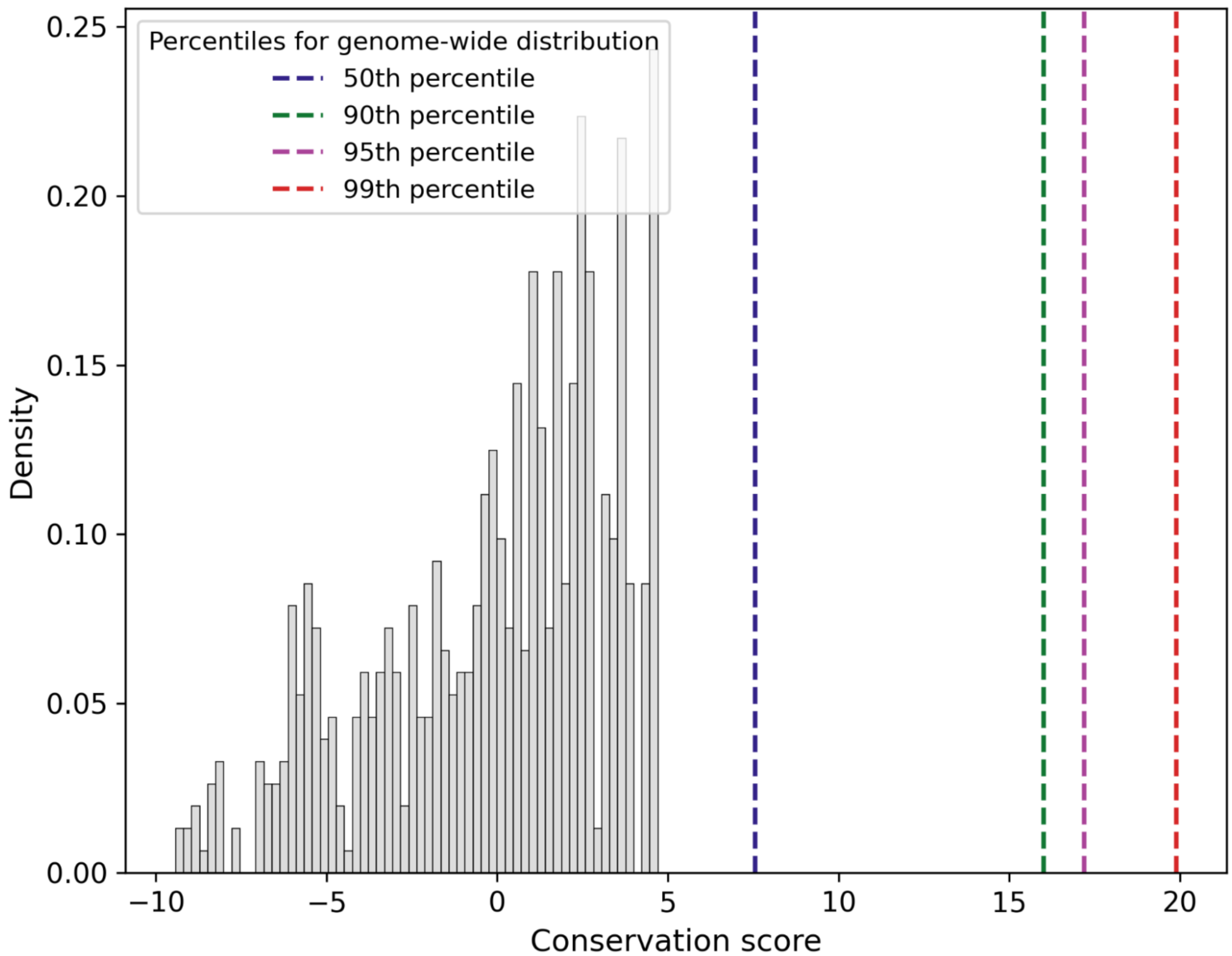

Fig. S14: Distribution of GERP scores for the gene *pvalb4*

Supplementary Tables

Table S1: Gill DEGs found in Rabbit Slough vs Cheney Lake F_{ST} outliers

Gene name	Gene ID	Chromosome	FST outliers	Tempo SNPs
fosb	ENSGACG00000009792	chrI	2	
cahz	ENSGACG00000014774	chrIII	22	
col6a1	ENSGACG00000012576	chrIII	18	
cmah	ENSGACG00000019485	chrIV	49	
tnmd	ENSGACG00000017466	chrIV	3	
hbegfa	ENSGACG00000018355	chrIV	29	
antxr1c	ENSGACG00000004906	chrV	1	
ENSGACG00000002171	ENSGACG00000002171	chrVIII	1	
ptgs2b	ENSGACG00000007384	chrVIII	21	
col11a2	ENSGACG00000000099	chrX	1	
pvalb4	**ENSGACG00000012174**	**chrXI**	**144**	**30**
tnnt1	ENSGACG00000006626	chrXI	7	
mylpfa	ENSGACG00000013640	chrXI	1	
cacnb1	ENSGACG00000009291	chrXI	139	
itga5	ENSGACG00000010945	chrXII	30	
si:dkey-205h13.1	ENSGACG00000011044	chrXII	20	
bgnb	ENSGACG00000010078	chrXII	10	
si:dkey-245n4.2	ENSGACG00000010369	chrXIII	10	
ENSGACG00000006275	ENSGACG00000006275	chrXVI	1	
ldha	ENSGACG00000011270	chrXIX	3	

rbm24b	ENSGACG00000008472	chrXX	6	
hsc70	ENSGACG00000007569	chrXX	12	
gapdh	ENSGACG00000010219	chrXX	17	
pi15a	ENSGACG00000002723	chrXXI	19	
Total				

Table S2: Gill DEGs found in Rabbit Slough vs Cornelius Lake F_{ST} outliers

Gene name	Gene ID	Chromosome	F_{ST} outliers	Tempo SNPs
col8a1b	**ENSGACG00000014373**	**chrI**	**227**	**40**
col6a1	ENSGACG00000012576	chrIII	7	
nxpe3	ENSGACG00000019508	chrIV	2	
cmah	ENSGACG00000019485	chrIV	30	
tmem168a	**ENSGACG00000018918**	**chrIV**	**57**	**18**
pcolcea	ENSGACG00000020298	chrVII	79	
S100P	ENSGACG00000020061	chrVII	1	
si:ch211-156l18.7	**ENSGACG00000018299**	**chrIX**	**79**	**13**
sod3b	**ENSGACG00000017840**	**chrIX**	**93**	**26**
myoz2b	ENSGACG00000017804	chrIX	116	
slc25a4	ENSGACG00000017708	chrIX	85	
ST3GAL1	ENSGACG00000008844	chrX	1	
pvalb4	**ENSGACG00000012174**	**chrXI**	**94**	**28**
ENSGACG00000005444	ENSGACG00000005444	chrXI	1	
pvalb8	**ENSGACG00000012178**	**chrXI**	**88**	**27**
ENSGACG00000012669	ENSGACG00000012669	chrXI	2	
ENSGACG00000012690	ENSGACG00000012690	chrXI	4	
si:dkey-205h13.1	**ENSGACG00000011044**	**chrXII**	**284**	**59**
itga5	ENSGACG00000010945	chrXII	1	
bgnb	ENSGACG00000010078	chrXII	1	

si:dkey-245n4.2	**ENSGACG00000010369**	**chrXIII**	**124**	**8**
nol6	**ENSGACG00000010296**	**chrXIII**	**312**	**8**
ldha	ENSGACG00000011270	chrXIX	7	
atp6v1e1a	ENSGACG00000013695	chrXIX	3	
calb2a	ENSGACG00000003013	chrXIX	2	
celf3b	ENSGACG00000011707	chrXX	185	
tubb5	ENSGACG00000009635	chrXX	5	
hsc70	**ENSGACG00000007569**	**chrXX**	**184**	**35**
hgd	**ENSGACG00000002559**	**chrXXI**	**429**	**8**

Table S3:Brain DEGs found in only Rabbit Slough vs Cheney Lake F_{ST} outliers

Gene name	Gene ID	Chromosome	FST outliers	Tempo SNPs
MAP4K1	ENSGACG00000013172	chrI	4	
cahz	ENSGACG00000014774	chrIII	22	
col6a1	ENSGACG00000012576	chrIII	18	
slc7a11	ENSGACG00000019609	chrIV	14	
CPNE8	ENSGACG00000019027	chrIV	28	
strip2	ENSGACG00000018938	chrIV	13	
foxp2	ENSGACG00000018911	chrIV	4	
ENSGACG00000018550	ENSGACG00000018550	chrIV	4	
ENSGACG00000018361	**ENSGACG00000018361**	**chrIV**	**161**	**27**
n4bp3	**ENSGACG00000018359**	**chrIV**	**105**	**40**
hbegfa	ENSGACG00000018355	chrIV	29	
ENSGACG00000018050	ENSGACG00000018050	chrIV	3	
DOCK2	**ENSGACG00000017885**	**chrIV**	**216**	**39**

ponzr1	ENSGACG00000019324	chrVII	4	
pnp5a	ENSGACG00000018988	chrVII	2	
myo18aa	ENSGACG00000020305	chrVII	7	
ENSGACG00000020069	ENSGACG00000020069	chrVII	19	
S100P	ENSGACG00000020061	chrVII	8	
ENSGACG00000018560	ENSGACG00000018560	chrVII	2	
f11r.1	ENSGACG00000020422	chrVII	17	
ENSGACG00000007974	ENSGACG00000007974	chrVIII	5	
slc25a24	ENSGACG00000006388	chrVIII	2	
b3gnt3.4	ENSGACG00000008643	chrVIII	11	
ENSGACG00000002171	ENSGACG00000002171	chrVIII	1	
add3a	ENSGACG00000018717	chrIX	3	
capn2b	ENSGACG00000019944	chrIX	2	
ENSGACG00000018430	ENSGACG00000018430	chrIX	1	
ENSGACG00000006358	ENSGACG00000006358	chrX	1	
ENSGACG00000014556	ENSGACG00000014556	chrXI	1	
slc4a1a	ENSGACG00000009622	chrXI	8	
dhx58	**ENSGACG00000008740**	**chrXI**	**153**	**80**
PKP1	ENSGACG00000009752	chrXII	19	
galnt6	ENSGACG00000008590	chrXII	1	
si:dkey-205h13.1	ENSGACG00000011044	chrXII	20	
ENSGACG00000011026	ENSGACG00000011026	chrXIII	4	
ENSGACG00000006275	ENSGACG00000006275	chrXVI	1	

rhag	ENSGACG00000009865	chrXVIII	1	
ENSGACG00000011188	ENSGACG00000011188	chrXIX	4	
nbeal2	ENSGACG00000008637	chrXX	14	
ENSGACG00000006045	ENSGACG00000006045	chrXX	26	
ncam3	ENSGACG00000005994	chrXX	7	
ENSGACG00000012665	ENSGACG00000012665	chrXX	1	
stmn1b	ENSGACG00000007379	chrXX	9	
tmod4	ENSGACG00000013516	chrXX	1	
arfgef1	ENSGACG00000002879	chrXXI	85	
tram1	ENSGACG00000002818	chrXXI	46	
cmbl	ENSGACG00000002636	chrXXI	37	

Table S4:Brain DEGs found in only Rabbit Slough vs Cornelius Lake F_{ST} outliers

Gene name	Gene ID	Chromosome	F_{ST} outliers	Tempo SNPs
arrb1	ENSGACG00000004570	chrI	21	
ENSGACG00000018030	**ENSGACG00000018030**	**chrIV**	**125**	**18**
slc6a1l	ENSGACG00000019748	chrIV	29	
slc7a11	ENSGACG00000019609	chrIV	1	
pfkfb3	ENSGACG00000019176	chrIV	1	
SYN3	ENSGACG00000019125	chrIV	350	
strip2	**ENSGACG00000018938**	**chrIV**	**100**	**5**
ITM2A	**ENSGACG00000018559**	**chrIV**	**131**	**55**
acsl4a	**ENSGACG00000018503**	**chrIV**	**274**	**82**
ENSGACG00000018050	ENSGACG00000018050	chrIV	8	
vamp2	ENSGACG00000020297	chrVII	62	
ENSGACG00000020330	**ENSGACG00000020330**	**chrVII**	**125**	**79**
ENSGACG00000020069	**ENSGACG00000020069**	**chrVII**	**217**	**102**
cacng1b	ENSGACG00000012215	chrXI	1	
dhx58	**ENSGACG00000008740**	**chrXI**	**50**	**31**
si:dkey-205h13.1	**ENSGACG00000011044**	**chrXII**	**284**	**59**
csad	ENSGACG00000009463	chrXII	12	
si:dkey-245n4.2	**ENSGACG00000010369**	**chrXIII**	**124**	**8**
ENSGACG00000008429	ENSGACG00000008429	chrXIII	23	
ENSGACG00000012351	ENSGACG00000012351	chrXX	3	
arfgef1	**ENSGACG00000002879**	**chrXXI**	**795**	**13**
gdap1	**ENSGACG00000002727**	**chrXXI**	**303**	**2**
cmbl	**ENSGACG00000002636**	**chrXXI**	**342**	**6**

Table S5: Missense variants associated with DEGs

Chromosome	Position	Ref	Alt	Gene
**Rabbit Slough vs Cheney Brain DEGs**				
chrIV	13353322	A	G	*n4bp3*

chrIV	13373668	T	A	
chrIV	13373744	T	C	
chrXI	6629831	A	T	*kat2a*
chrXI	6636856	A	C	*dhx58*
chrXI	6637178	G	A	*dhx58*
chrXI	6639650	A	G	*dhx58*
**Rabbit Slough vs Cornelius Brain DEGs**				
chrIV	11016657	T	G	*myoz3a*
chrIV	15071531	C	G	*acsl4a*
chrIV	15073110	C	A	*acsl4a*
chrIV	15073356	A	G	*acsl4a*
chrIV	15073383	A	G	*acsl4a*
chrIV	15076140	T	A	*acsl4a*
chrIV	15745378	T	G	*gpr174*
chrIV	15745501	T	C	*gpr174*
chrIV	15745531	A	C	*gpr174*
chrIV	15745773	T	G	*gpr174*
chrIV	15745775	T	C	*gpr174*
chrIV	26923560	T	C	*strip2*
chrVII	10849566	G	A	*tbc1d14*
chrVII	10858813	C	A	
chrXI	6636856	A	C	*dhx58*
chrXII	6616188	G	A	*si:dkey-205h13.1*

chrXIII	10698847	T	G	*aqp3a*
**Rabbit Slough vs Cornelius Gill DEGs**				
chrI	26106349	C	G	*col8a1b*
chrI	26106391	T	G	*col8a1b*
chrI	26106589	G	T	*col8a1b*
chrI	26106634	G	C	*col8a1b*
chrI	26106679	T	C	*col8a1b*
chrI	26106727	T	A	*col8a1b*
chrI	26106956	C	A	*col8a1b*
chrI	26107096	T	C	*col8a1b*
chrI	26107789	G	C	*col8a1b*
chrI	26108583	A	T	*col8a1b*
chrI	26108590	T	C	*col8a1b*
chrI	26108617	T	C	*col8a1b*
chrI	26108692	C	T	*col8a1b*
chrIX	11316900	G	C	*si:ch211-156l18.7*
chrIX	11318337	C	T	*si:ch211-156l18.7*
chrIX	13523523	C	G	*sod3b*
chrIX	13523602	A	G	*sod3b*
chrIX	13523614	A	G	*sod3b*
chrIX	13527072	C	T	*DHX15_(1_of_many)*
chrIX	13528335	T	C	*DHX15_(1_of_many)*
chrXII	6616188	G	A	*si:dkey-205h13.1*

chrXIII	10698847	T	G	*aqp3a*
chrXX	10614960	G	A	*lin37*
chrXX	10618297	G	C	*si:ch211-137j23.7*
chrXX	10618381	A	G	*si:ch211-137j23.7*
chrXX	10618408	A	G	*si:ch211-137j23.7*
chrXX	10618602	T	C	*si:ch211-137j23.7*
chrXX	10618636	C	A	*si:ch211-137j23.7*
chrXX	10618651	G	A	*si:ch211-137j23.7*
chrXX	10623887	G	T	*zgc:171592*

References

Roberts Kingman, Garrett A., Deven N. Vyas, Felicity C. Jones, et al. 2021. "Predicting Future from Past: The Genomic Basis of Recurrent and Rapid Stickleback Evolution." *Science Advances* 7 (25). https://doi.org/10.1126/sciadv.abg5285.
